# Fibrotic Scars and the Potential Therapeutics After Spinal Cord Injury

**DOI:** 10.3390/cells15211977

**Published:** 2026-10-30

**Authors:** Ching-Yi Lin, Alex Nelson, Jason Lin, Wayne Lee, Sidney Bihrer, Yu-Shang Lee

**Affiliations:** 1 Department of Neurosciences, Cleveland Clinic Research, Cleveland, OH 44195, USA; 2 Cleveland Clinic Lerner College of Medicine, Case Western Reserve University School of Medicine, Cleveland, OH 44195, USA

**Keywords:** spinal cord injury (SCI), fibrotic scars, fibrosis, extracellular matrix (ECM), fibronectin, fibroblasts, neuroregeneration

## Abstract

Spinal cord injury (SCI) affects millions of people worldwide, resulting in persistent direct and secondary effects including but not limited to motor, sensory, and autonomic dysfunction. The widespread nature of this condition, as well as its detrimental effects, makes recovery following SCI an important clinical problem. One primary issue identified in SCI among other neurological conditions is fibrotic scarring, as its dynamic nature provides both beneficial and detrimental effects over its developmental course. Initially, early scar formation contributes to wound closure and tissue stabilization, but chronic, persistent fibrosis later interferes with axonal growth and other neural repair mechanisms. The scar itself is highly dynamic, consisting of components including collagens, fibronectin, proteoglycans, and laminins, all of which have diverse localization and functionality throughout scar maturation. Its formation is regulated by various signaling mechanisms, including inflammatory signaling, profibrotic pathways, vascular responses, interactions between fibroblasts and neighboring cells, and changes in the specific mechanical environment of the injury site. This review focuses primarily on SCI, with findings from additional central nervous system injury and disease models used to provide a holistic, clinically relevant overview of fibrotic scar organization, profibrotic signaling, and potential therapeutic targets.

## 1. Introduction

Spinal cord injury (SCI) presents as a major cause of long-term disability, affecting an estimated 15.4 million people worldwide and accounting for more than 4.5 million years lived with disability as of 2021 [1]. Depending on the level and severity of the injury, persistent motor, sensory, and autonomic dysfunction can result, leading to secondary complications and long-term rehabilitation needs that affect both the individual and society as a whole. These chronic consequences emphasize the importance of identifying and studying SCI recovery as a prominent clinical problem, providing a strong rationale for understanding the underlying mechanisms and signaling supporting early repair through fibrosis while also limiting later neural regeneration. All abbreviations and gene symbols used are summarized at the end of the text.

## 2. Composition and Organization of the Fibrotic Scar Extracellular Matrix

Following SCI, the fibrotic scar develops around a dense extracellular matrix (ECM) composed primarily of collagens, fibronectin, proteoglycans, and laminins, with the matrix continually changing as the scar matures. Its composition, macro-organization, and mechanical properties shift over time, simultaneously impacting fibroblast behavior and the environment encountered by injured axons at the lesion. Much of what is known about these changes comes directly from SCI models, with supplemental mechanistic insight coming from other in vivo and in vitro systems [2,3,4,5,6].

The fibroblast populations responsible for producing this matrix are not a uniform group, as seen through the various approaches taken to characterize them. To clarify the cell types involved in the fibrotic response, an early study conducted by Göritz et al. used glutamate-aspartate transporter (GLAST)-CreER lineage tracing, a tamoxifen-inducible system in which Cre recombinase is fused to a modified estrogen receptor ligand-binding domain, to identify a perivascular population termed type A pericytes that expanded after SCI and gave rise to scar-forming cells [7]. Immunostaining showed cells were positive for platelet-derived growth factor receptor beta (PDGFRβ) and cluster of differentiation 13 (CD13), both of which are commonly expressed by pericytes. Subsequent ultrastructural analysis placed them within the vascular basal lamina, further supporting their original classification as pericytes. Later, Soderblom et al. identified *Col1a1*-expressing perivascular fibroblasts as a major source of the fibrotic scar following contusive SCI with an intact dura [3]. The authors used a *Col1a1*-green fluorescent protein (GFP) reporter to identify cells with active *Col1a1* promoter activity and separately used neuron-glial antigen 2 (NG2)-CreER lineage tracing to show that these fibroblasts were distinct from NG2-lineage pericytes. However, neither marker is unique to this population; PDGFRβ and CD13 are also expressed by other perivascular populations, including *Col1a1*-expressing fibroblasts and other pericytes, while PDGFRβ is also shared by vascular smooth muscle cells (vSMCs) [3,5,7].

Dias et al. then examined the importance of the GLAST-lineage type A pericytes using graded attenuation of Ras signaling [8]. Moderate attenuation reduced fibrosis and improved axonal regeneration and sensorimotor recovery, with measurable outcomes including fewer hindlimb placement errors and better inter-limb coordination. Near-complete suppression, however, was found to impair lesion closure, indicating that scar formation is required to some degree for wound repair. Still, it remains unclear how these GLAST-lineage cells corresponded to the *Col1a1*-expressing fibroblasts described in other lineage models. Both populations were associated with the spinal cord vasculature and expressed PDGFRβ and CD13, with Soderblom et al. identifying these markers in 95.7% and 78.7% of *Col1a1*-GFP+ cells, respectively [3,7]. Since these markers are expressed by both populations, they cannot be used alone to determine whether the cells share a common lineage or represent separate perivascular populations. The methods used to identify these populations also differed, as Göritz et al. used GLAST-CreER lineage tracing to follow descendants of cells labeled before injury, whereas Soderblom et al. used a *Col1a1*-GFP reporter to identify cells expressing Col1a1 and NG2-CreER lineage tracing to uncover the contribution of NG2^+^ pericytes [3,7]. While the NG2-lineage cells were distinguishable from the *Col1a1*-expressing fibroblasts, Soderblom et al. acknowledged that only a portion of the NG2^+^ pericytes were labeled by their tracing system. Therefore, the absence of substantial overlap between these labeled populations does not necessarily exclude other pericyte populations from contributing to the fibrotic scar. The authors also suggested that the *Col1a1*-expressing fibroblasts could represent the same population as the GLAST-lineage type A pericytes described by Göritz et al., with the NG2^+^ pericytes potentially corresponding to type B pericytes. However, neither study directly compared these populations using the same lineage-tracing approach, so the extent to which they represent the same cells remains unknown. The differences in the lineage-tracing methods, cellular markers, and injury models used across these studies are summarized in Table 1.

More recently, single-cell RNA sequencing (scRNA-seq) and lineage-tracing studies have helped clarify which cells generate the ECM components of the fibrotic scar. To manage the overlap in PDGFRβ expression among fibroblasts, pericytes, and vSMCs, Xue et al. combined scRNA-seq with several independent distinguishing lineage systems: *Col1a2*-CreER for fibroblasts, *Myh11*-CreER and NG2-CreER for pericytes/vSMCs, and *Crabp2*-CreER for meningeal fibroblasts [5]. To further examine the differences between these populations, Xue et al. used several CreER mouse lines crossed with Rosa26-tdTomato reporter mice, allowing them to trace the contributions of different cellular populations following SCI. Specifically, this system uses Cre recombinase attached to a modified estrogen receptor, which allows recombination to be controlled through the administration of tamoxifen. Following treatment, Cre removes a stop sequence within the Rosa26 reporter, resulting in permanent tdTomato expression in the targeted cells as well as their descendants. This allowed the researchers to examine whether cells labeled before injury were later present within the fibrotic scar, rather than identifying their origins based solely on the expression of individual cellular markers. Using this approach, different CreER lines were used to examine the contributions of fibroblasts, pericytes, and vascular smooth muscle cells following both transection and crush SCI. In PDGFRβ-CreER mice, less than 8% of the labeled cells also expressed desmin after injury, indicating that PDGFRβ was not specific to desmin-positive pericytes and vSMCs. When examining the *Myh11*-CreER mice, the researchers found that the majority of labeled cells remained associated with the blood vessels, with relatively few cells observed throughout the collagen-rich fibrotic lesion. A similar distribution was observed using NG2-CreER tracing, with the labeled pericyte and vascular smooth muscle cell populations contributing relatively little to scar formation. However, when using *Col1a2*-CreER to trace fibroblasts, these cells were found throughout the collagen-positive lesion, supporting the conclusion that fibroblasts were the primary contributors to fibrotic scar formation rather than the pericyte and vascular smooth muscle cell populations examined in these injury models [5]. Xue et al. also observed transient increases in *Acta2* and *Tagln* expression among fibroblasts at 5 days post-injury, suggesting that these cells underwent changes associated with myofibroblast activation, rather than necessarily representing another cellular lineage. The specific fibroblast populations and distribution were also heterogeneous. Meningeal-derived *Crabp2^+^/Emb^+^* fibroblasts (CE-F) localized primarily to the central scar and showed higher *Col1a1* and *Fn1* expression, while perivascular and pial *Lama1^+^/Lama2^+^* fibroblasts (LA-F) were mostly found toward the lesion periphery and showed stronger laminin and type IV collagen expression. A similar distinction was observed in monkey SCI, suggesting that this fibroblast heterogeneity is not restricted to rodent injury models [5]. A comparison of the experimental approaches and findings from these studies is provided in Table 1.

### 2.1. Collagen and Laminin Provide Structural and Region-Specific Organization of the Fibrotic Scar

Collagen increasingly accumulates during scar maturation, while the distribution of different collagen and laminin components differs across the fibrotic site. CE-F populations show higher *Col1a1* expression, while LA-F populations show higher *Lama1/Lama2* and *Col4a1* expression [5]. This result is consistent with studies classifying the structural roles of these ECM components: type I collagen assembles into fibrils that provide structural and mechanical support, whereas type IV collagen forms interconnected networks within basement membranes [9,10]. Similarly, Colognato et al. demonstrated that laminin polymerizes into a network along muscle cell surfaces, a process associated with basement membrane assembly [11].

Fibrillar collagen accumulation also develops alongside major mechanical changes within the injured spinal cord. Using second-harmonic generation microscopy to examine fibrillar collagen and atomic-force microscopy to measure tissue stiffness, Manesco et al. found that fibrillar collagen progressed toward a denser and more tortuous state as the scar matured [12]. Specifically, the lesion was measured to be relatively soft soon after injury but stiffened substantially over the following weeks, coinciding with approximately the same time organized fibrillar collagen became prominent at the injury site. Although the two processes follow a similar timeline, collagen not being selectively manipulated in this study restricts the causal nature of the results. How these changes in matrix mechanics may feed back onto fibroblast activation is discussed later with the factors that regulate fibrotic scar formation.

### 2.2. Fibronectin Supports Matrix Assembly and Persistence During Scar Maturation

Fibronectin appears early in the lesion, before a mature fibrotic matrix has formed. At 3 days after SCI, fibronectin is distributed diffusely throughout the injury site despite the presence of relatively few fibroblasts [6]. The authors proposed that much of this early pool may represent plasma fibronectin entering through disruption of the blood–spinal cord barrier. By 7 days, the pattern changes: fibroblasts and macrophages/microglia are abundant, and fibronectin becomes considerably more condensed and fibrillar. Biochemical fractionation shows the same transition, with detergent-insoluble fibronectin, indicative of a more stably assembled matrix, increasing markedly at 7 and 14 days.

Production of fibronectin is only one part of building this matrix. Integrin α5β1, the canonical fibronectin receptor, increases after SCI and is expressed by fibroblasts as well as activated macrophages and microglia, with particularly high expression in the myeloid population [6]. Despite this increased expression, deletion of fibronectin from lysozyme M (LysM)-lineage myeloid cells had little effect on overall lesion fibronectin, indicating that these cells are not the primary source.

Beyond production, soluble fibronectin initially seen at the injury site must be assembled into fibrils to form stable polymers. In cultured fibroblasts, Rho-dependent actomyosin tension allows cells to pull on extracellular fibronectin and expose cryptic self-association sites that promote fibril assembly [13]. Furthermore, fluorescence resonance energy transfer similarly demonstrated that force generated by the cell stretches fibronectin, thereby changing the accessibility of binding sites within its type III domains [14]. Fibronectin matrix formation therefore depends on both protein availability and the cellularly generated mechanical forces.

Once assembled, fibronectin supports the formation of the collagen-rich matrix. In fibroblast culture, type I and III collagen continue to be synthesized in the absence of fibronectin, but organization into a coherent extracellular network is greatly reduced. Adding soluble fibronectin provides the materials required for a fibronectin network, leading to formation of the fibronectin matrix followed by more organized collagen deposition [15]. This association has not been demonstrated directly after SCI, so fibronectin cannot yet be considered a tested requirement for collagen assembly in the spinal scar. Still, the culture findings provide a basis for how an early fibronectin-rich matrix could influence later collagen organization.

As with the other components, the composition of the fibronectin matrix also changes over time. Cooper et al. examined fibronectin containing alternatively spliced Extra Domain A (FnEDA) and found that loss of this region had relatively little effect on the early fibronectin response [16]. The differences became more apparent during later scarring stages, where FnEDA-null mice began to show reductions in both insoluble fibronectin and overall chronic fibrosis. This later reduction suggests that FnEDA contributes more to persistence of the chronic fibronectin matrix than to its acute formation.

### 2.3. Proteoglycans Contribute to Matrix Organization and Regenerative Inhibition

Chondroitin sulfate proteoglycans (CSPGs) consist of two main structural parts: a protein core with covalently attached chondroitin sulfate (CS) glycosaminoglycan (GAG) chains, referred to here as CS-GAG chains. Although variation exists between components comprising individual CSPGs, there are two overall portions of the molecule that uniquely contribute to observable interactions with the surrounding microenvironment. For example, CS-GAG chains contribute directly to binding with the neuronal receptor protein tyrosine phosphatase sigma (PTPσ), while the G3 domain of the versican protein core can interact with other structural molecules like collagen [17,18]. Following SCI, CSPGs are distributed across several lesion compartments spanning both the fibrotic and glial scar, as both compartments showed strong CSPG immunoreactivity 2 weeks after contusive SCI [19]. Quantitatively, immunohistochemical studies examining CSPGs containing chondroitin-6-sulfate showed a rise in immunoreactivity within the first week after rat contusion SCI and remained elevated for at least 40 days, demonstrating the persistent elevation of CSPGs throughout scar maturation [20].

Looking at total abundance eclipses differences in localization and temporal variation among individual CSPG types. Jones et al. examined several CSPGs following dorsal-column SCI, where they found neurocan, brevican, and versican increased within several days and reached maximal immunoreactivity at approximately 2 weeks [21]. Furthermore, neurocan and versican remained elevated through roughly 4 weeks, whereas brevican remained elevated for at least 2 months. However, phosphacan followed a different pattern, decreasing in tissue immediately adjacent to the lesion early after injury before recovering and reaching its highest levels later. The study also provided evidence for the cellular source of one of these CSPGs, with in situ hybridization identifying reactive glial fibrillary acidic protein (GFAP)-positive astrocytes as a source of neurocan in this model. Overall, the CSPG response changes over time in accordance with the proteoglycan species being measured.

As previously introduced, versican has an additional structural role through its interactions with collagen. Although it has been confirmed present around spinal lesions and within fibroblast grafts, its effects on ECM organization have mainly been studied outside the central nervous system (CNS). Chen et al. examined this relationship using fibroblast-derived matrices, collagen-versican gels, and tissue-based models [18]. In NIH 3T3 fibroblast-derived matrices, the presence of versican increased collagen deposition and shifted the fibers toward a more highly aligned architecture. Solid-phase binding assays further showed that this interaction occurred primarily through the G3 domain of the versican core protein. The presence of versican also altered the mechanical behavior of purified collagen gels, namely through decreases in baseline shear stiffness and weakening of the normal stiffening response under increasing shear strain. Similarly, their rat liver tissue studies showed disruption of either versican or its GAG chains altered compression-dependent stiffening. These findings provide several possible mechanisms through which versican could influence collagen organization and matrix mechanics, which could be examined directly in future SCI studies.

### 2.4. Fibrotic ECM Composition and Organization Evolve During Scar Maturation

**Figure 1:**
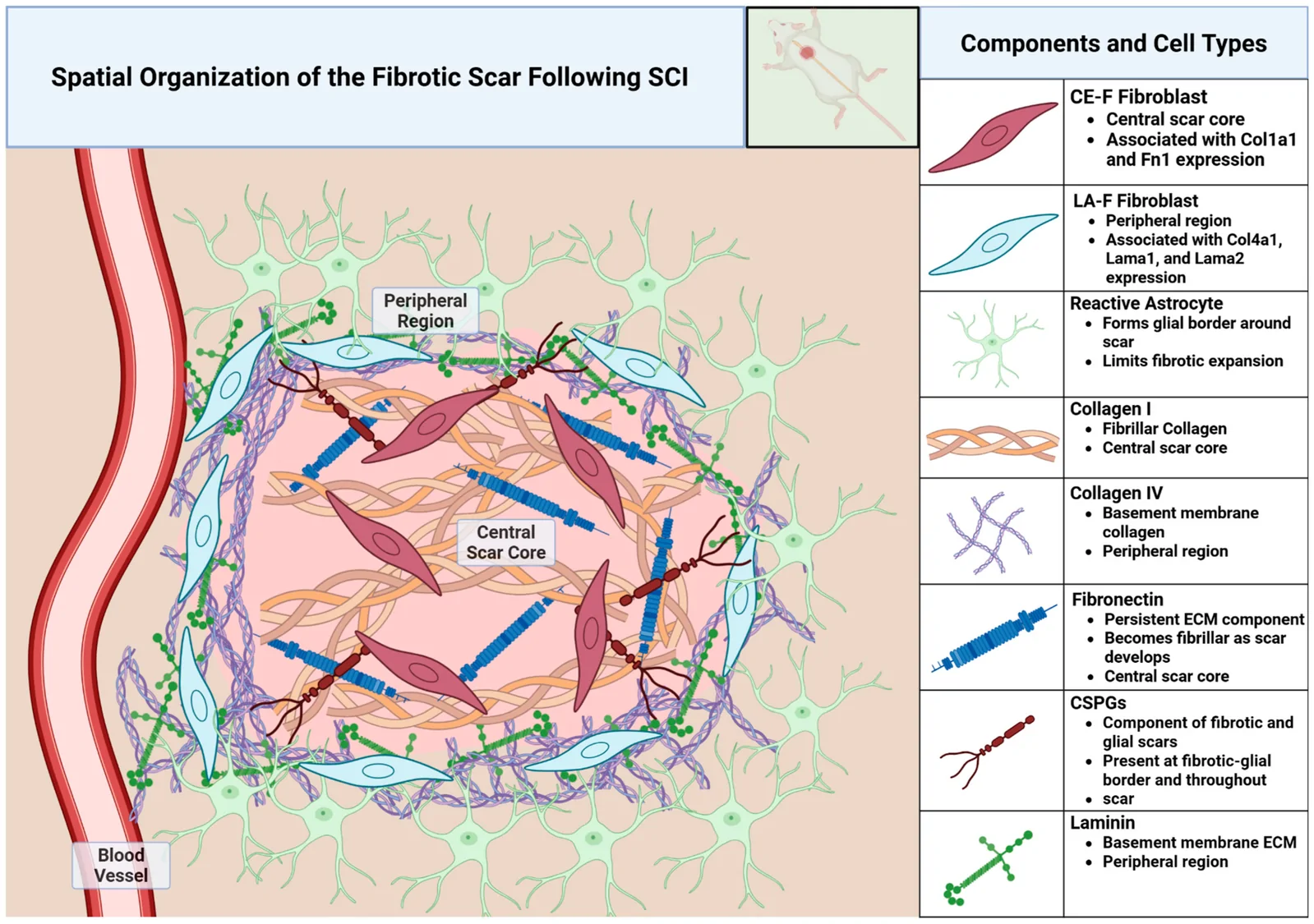
Major extracellular matrix components and their spatial distribution within the fibrotic scar following spinal cord injury (SCI). The associated diagram demonstrates the central scar core, lesion periphery, and surrounding astrocytic border. The meningeal-derived *Crabp2^+^*/*Emb^+^* fibroblast (CE-F) population has higher *Col1a1* and *Fn1* gene expression and is concentrated in the central scar core, consistent with the enrichment of collagen I (fibrillar collagen) and fibronectin in this region. Organizationally, fibronectin initially presents as diffuse and soluble but eventually becomes more condensed and fibrillar as the scar matures. The perivascular/pial *Lama1^+^*/*Lama2^+^* fibroblast (LA-F) population migrates to the lesion periphery from perivascular and pial sources, with the perivascular association represented by the blood vessel in the illustration. LA-Fs predominantly express *Col4a1*, *Lama1*, and *Lama2*, consistent with the localization of collagen IV (basement membrane collagen) and laminin around the peripheral region of the scar. Additionally, chondroitin sulfate proteoglycans (CSPGs) are found across the fibrotic and glial scar compartments, with different CSPGs having distinct spatial and temporal distributions. Astrocytes form the glial border surrounding the fibrotic compartment, limiting the spread of fibrotic and inflammatory cells into surrounding neural tissue. Overall, the spatial depictions represent the expected regional enrichment of each component but do not indicate that these components are restricted exclusively to the depicted regions.

Scar maturation involves more than simply increasing the amount of ECM present at the lesion. Early fibronectin shifts from a diffuse and largely soluble pool into a more stable fibrillar matrix, while fibrillar collagen becomes increasingly prominent alongside changes in lesion stiffness. Individual CSPGs also follow different timelines rather than increasing uniformly throughout scar development. These changes occur spatially as well, with CE-F and LA-F populations occupying different regions of the lesion and favoring different ECM components (Figure 1). Therefore, the chronic fibrotic scar is not just a denser version of the acute lesion, as its composition and organization continue to change throughout wound repair.

## 3. Context-Dependent Functional Consequences of Fibrotic Scarring

Following CNS injury, fibrotic scar formation is associated with both beneficial and detrimental effects, with the balance between these functions contributing to the final outcome. Early in the fibrotic response, scar-forming cellular populations form and interact with the ECM, initially helping to restore continuity across disrupted tissue. Over time, persistence of this response can interfere with axonal regrowth and other pathways of neural regeneration. These effects are context-dependent, varying based on the magnitude and timing of fibrosis, the composition of the ECM at that specific stage, and interactions between the fibrotic scar and surrounding neural, glial, and immune cells. Direct SCI studies provide clear evidence for both the requirement of fibrosis during wound closure and its later restriction of regeneration, while studies in other disease models like stroke and inflammatory demyelination provide additional evidence for effects on inflammation and oligodendrocyte-lineage repair.

### 3.1. Early Fibrotic Scarring Supports Wound Closure and Lesion Compartmentalization

**Figure 2:**
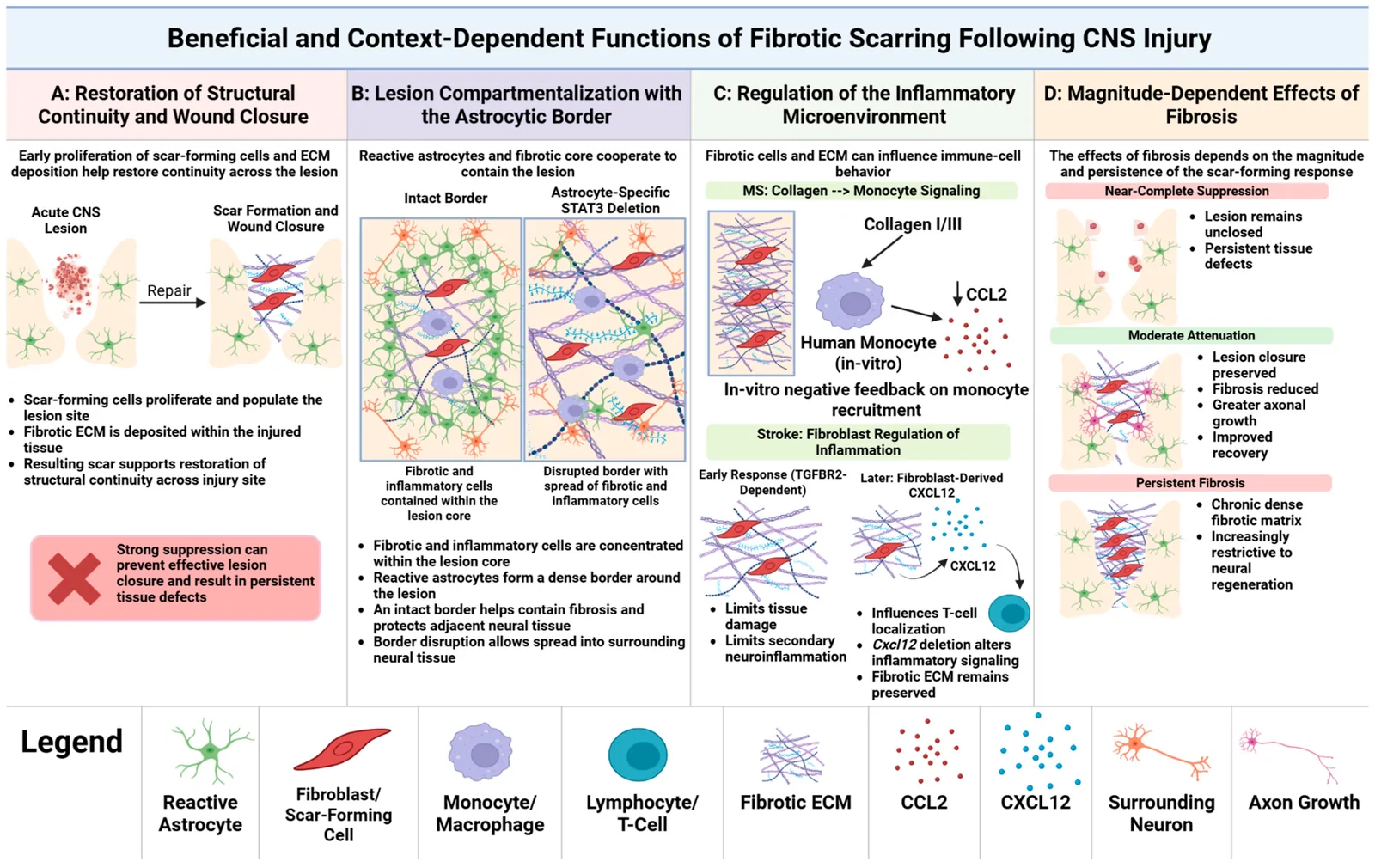
Beneficial and context-dependent functions of fibrotic scarring following central nervous system injury. In early stages of scar formation, reparative effects include wound closure, lesion compartmentalization, and regulation of inflammatory factors. However, the degree of benefit depends on the magnitude and persistence of the fibrotic response. (**A**) Within SCI, glutamate-aspartate transporter (GLAST)-lineage scar-forming cells originally termed type A pericytes expand after injury and contribute to scar formation. Suppressing this proliferation strongly enough prevents effective wound closure and leaves persistent tissue defects across the injury site. Through graded attenuation, it was further shown that near-complete suppression impaired wound closure while moderate attenuation reduced fibrosis without preventing restoration of tissue integrity. (**B**) Reactive astrocytes form a dense, signal transducer and activator of transcription 3 (STAT3)-dependent border around the fibrotic compartment, containing fibroblastic and inflammatory cells within the lesion core. Astrocyte-specific STAT3 deletion was shown to disrupt this border formation, leading to fibrotic and inflammatory cells spreading further into surrounding neural tissue, accompanied by increased neuronal loss. The benefit, therefore, results primarily from compartmentalization of the fibrotic and inflammatory response rather than a direct protective effect of the fibrotic extracellular matrix (ECM) itself. (**C**) In human multiple sclerosis (MS), perivascular regions of inflammatory lesions contain collagens I, III, and V, as well as biglycan and decorin. Human monocytes exposed to fibrillar collagen I/III in vitro had reduced C-C motif chemokine ligand 2 (CCL2) production. Since CCL2 promotes monocyte recruitment, this could provide negative feedback on continued inflammatory-cell recruitment, although this effect has not been demonstrated in vivo. Ischemic stroke further shows temporal differences in fibroblast function. Early on, the fibrotic response was transforming growth factor beta receptor 2 (TGFBR2)-dependent, as fibroblast-specific deletion of *Tgfbr2* impaired fibrotic lesion formation and resulted in greater cortical tissue loss and markedly increased mortality. Through this, it was shown that an intact myofibroblast response is necessary to limit early-to-subacute ischemic brain damage. Later stages showed C-X-C motif chemokine ligand 12 (CXCL12) as an important fibroblast-derived signaling factor involved in T-cell localization, as fibroblast-specific deletion of *Cxcl12* altered T-cell localization and resulted in elevated inflammatory cytokine expression. Importantly, fibroblastic ECM deposition remained largely preserved, helping to separate this immune-organizing function from fibrotic ECM deposition. (**D**) The functional effects of fibrosis are strongly dependent on the magnitude of the scar-forming response. Moderate attenuation preserves lesion closure while reducing fibrosis and is accompanied by greater axonal growth and improved recovery. At the lower extreme, near-complete suppression prevents effective lesion closure and results in persistent tissue defects. At the same time, persistent fibrosis produces a chronic dense fibrotic matrix that becomes increasingly restrictive to neural regeneration. Therefore, early fibrosis can serve as an adaptive response, with its functional consequences depending on magnitude, timing, persistence, ECM context, and the specific injury model.

Looking past the misconception of fibrotic scarring solely serving as an impediment to neural regeneration, it is well-supported that initial formation plays an important wound-healing role after SCI. As previously described, type A pericytes (a GLAST-lineage population of scar-forming cells) expanded after injury and gave rise to many of the scar-forming cells within the lesion [7]. When scar-forming cells were experimentally generated from this perivascular population, the injured spinal cord failed to close properly, leaving persistent tissue defects at the lesion site [7]. These findings demonstrated that the contribution of this population to fibrotic scar formation was also important for restoring tissue integrity following SCI. It was later shown that the degree of fibrosis contributed to functional outcomes [8]. Animals in which the GLAST-lineage response was nearly eliminated similarly failed to close the lesion, whereas an intermediate reduction in proliferation reduced fibrosis without preventing restoration of tissue integrity. Therefore, some degree of fibrotic scar formation is required during early stages of wound repair, but excessive or persistent fibrotic production is not necessary for wound closure and can later become restrictive to regeneration [7,8] (Figure 2A,D).

Additionally, the fibrotic compartment works closely in conjunction with the surrounding astrocytic scar. These cell populations develop in close proximity following SCI, where fibroblastic and inflammatory cells become concentrated within the lesion core and newly proliferated reactive astrocytes form a dense border around them [22]. To test this relationship, Wanner et al. disrupted this border through astrocyte-specific deletion of signal transducer and activator of transcription 3 (STAT3), leading to the spread of fibrotic and inflammatory cells into neighboring neural tissue and an accompanying increase in neuronal loss. This experiment more directly demonstrates the protective function of the astrocytic border rather than the fibrotic ECM itself, but it shows that the spatial organization of the two scar compartments is important for wound repair and containment of fibrotic and inflammatory cells [22] (Figure 2B).

The fibrotic ECM itself can also influence immune-cell behavior after CNS injury. Mohan et al. found increased fibrillar collagens (I, III, and V), biglycan, and decorin in human multiple sclerosis inflammatory lesions [23]. Much of this matrix accumulated within perivascular spaces associated with infiltrating immune cells, although collagen V was also present in heavily infiltrated parenchyma of active lesions. In culture, fibrillar collagens I and III reduced production of C-C motif chemokine ligand 2 (CCL2) by human monocytes [23]. Since CCL2 promotes monocyte recruitment, collagen deposition could provide negative feedback on continued inflammatory-cell recruitment, although the applicability of this mechanism in vivo remains untested (Figure 2C).

A similar protective fibroblast response has also been observed after experimental brain ischemia. Ewing-Crystal et al. identified different fibroblast states throughout different stages following injury [24]. Early profibrotic myofibroblasts developed from resident brain fibroblasts and populated the lesion, whereas later fibroblast states showed greater interactions with lymphocytes. Fibroblast-specific deletion of *Tgfbr2* disrupted the early myofibroblast response, leading to greater tissue loss, secondary neuroinflammation, and substantially increased mortality following severe ischemic injury. Later in recovery, fibroblasts appeared to affect inflammation in a different way. Deletion of fibroblast-derived *Cxcl12* changed where T cells accumulated within the lesion and increased inflammatory cytokine signaling, including interferon gamma (IFN-γ) responses, while fibrotic ECM deposition remained preserved. Although these findings come from cerebral ischemia rather than directly from SCI, they support a broader protective role for fibroblast responses in limiting tissue damage and organizing inflammation after CNS injury (Figure 2C).

### 3.2. Persistent Fibrotic Scarring Restricts Axonal and Glial Repair

Although scarring initially plays an important function in wound closure, sustained persistence of the scar can lead to restrictions on long-term axonal regeneration. This effect can be seen in the previously mentioned Dias et al. study, where moderate attenuation of fibrotic scar formation reduced the number of dystrophic corticospinal tract (CST) endings and allowed injured CST axons to extend closer toward the injury site [8]. Dias et al. further examined the axonal response following moderate attenuation of fibrotic scar formation using biotinylated dextran amine (BDA) tracing to identify CST axons extending through the injury site. By four weeks, some CST axons were observed having entered and extended through the scar, with later time points showing further regeneration of fibers caudal to the injury. More specifically, 10.5 ± 2.1% of the traced CST axons entered the lesion, with some fibers extending as far as 4 mm caudal to the injury site. The authors also reported an absence of BDA-labeled fibers within the main dorsal CST below the lesion, helping rule out the possibility that the axons observed beyond the injury were simply spared fibers remaining from the initial injury. In addition to the anatomical tracing, Dias et al. examined whether these regenerated axons could functionally interact with the existing spinal circuitry. Synaptic markers were observed near traced CST terminals, while optogenetic stimulation of channelrhodopsin-2-expressing CST fibers generated postsynaptic responses in neurons below the lesion. Unlike studies where increased axonal density was the primary measure of axonal growth, these experiments provided both anatomical evidence of CST regeneration and electrophysiological evidence that the axons were capable of forming functional connections with spinal neurons following SCI. Interestingly, only 6.6 ± 3.1% of traced CST endings penetrated the glial-fibrotic interface far enough to contact yellow fluorescent protein-labeled GLAST-lineage scar-forming cells at two weeks, while larger populations instead encountered reactive astrocytes, oligodendrocyte progenitor cells, and cluster of differentiation 68 (CD68)-positive inflammatory cells before reaching the fibrotic core. Similarly, serotonergic raphespinal fiber density was increased below the lesion, although regeneration and sprouting of spared populations could not be effectively distinguished. Since attenuation also altered inflammation and reactive astrogliosis, these changes likely reflect modification of the broader lesion environment rather than removal of a single physical barrier, but the study overall provides evidence that reducing persistent fibrosis can improve the ability of injured axons to approach and cross the lesion [8].

A similar temporal progression of scar-derived inhibition is present in the deposition of fibronectin. Cooper et al. examined fibronectin containing the alternatively spliced FnEDA and found that elimination of FnEDA had relatively little effect on the acute fibrotic response [16]. However, the chronic fibrotic response experienced reduced detergent-insoluble fibronectin and overall fibrosis, indicating the importance of FnEDA in long-term stabilization of the fibronectin matrix in the ECM. This was further shown in the FnEDA-null animals, as they showed greater axonal density within the lesion and improved locomotor recovery, while the astrocytic scar remained largely unchanged. This separates the early formation of fibrotic tissue from stabilization of the chronic matrix and indicates that the later response can be reduced without preventing the initial scar from forming, suggesting that early stabilization of the injury site may be preserved while still allowing a more permissive environment for later neural regrowth. Cooper et al. examined the effects of FnEDA deletion on axonal density using SMI-312 immunostaining, which allowed them to identify neurofilament-positive axons within and surrounding the lesion at 12 weeks after SCI. However, it remained unclear whether the greater axonal density resulted from regeneration of injured axons or preservation of those that had not been severed by the injury. This distinction could not be made from SMI-312 staining alone, as the researchers did not use anatomical tracing to identify where the axons originated or determine whether they had grown through the lesion following SCI. While the reduction in chronic fibrosis was accompanied by increased axonal density and improved locomotor performance, the findings could not establish how much of the functional recovery was attributable to regeneration rather than preservation of existing axons [16].

A separate approach to reducing the fibrotic scar revolved around the reduction in hematogenous macrophages. Spatially, blood-derived macrophages become concentrated within the fibrotic core following SCI, while microglia remain more associated with the surrounding astroglial region. Zhu et al. demonstrated this in a contusion SCI mouse model through clodronate liposome-induced depletion, specifically targeting this cell population to separate its effects on scar modulation from that of microglia [25]. At both 7 and 14 days post-SCI, a reduction in cluster of differentiation 11b-positive (CD11b^+^) cells was observed, along with an accompanying reduction in fibroblast density. More specifically, the dense peripheral population of fibroblasts was reduced, along with basal-lamina formation between the fibroblasts and surrounding astrocytes. This variation in cellular makeup was not necessarily one-to-one with fibrotic scar reduction, as the scar area varied over the course of the experiment. Notably, the scar area was similar at 7 days, became larger at 14 days, and smaller by 56 days in the mice treated with clodronate. To determine whether this increase was related to additional axonal growth or simply a reduction in axonal retraction following injury, the authors compared the density of neurofilament-positive axons at 14 and 56 days after SCI. At 14 days, there was no significant difference in axonal density between the groups, whereas by 56 days, considerably more neurofilament-positive axons were observed within the lesion of the clodronate-treated animals. This later increase was interpreted by the authors as additional axonal growth rather than simply reduced axonal retraction. However, the neurofilament staining did not establish whether these additional axons were previously injured or originated from spared fibers, as the authors did not perform anatomical tract tracing to distinguish the two possibilities. Therefore, these findings support increased axonal growth following macrophage depletion, although regeneration of previously severed axons was not directly established. Despite the increased neurofilament-positive axons, the clodronate-treated mice did not show better open-field locomotor recovery based on the Basso Mouse Scale (BMS). Since macrophage depletion affects considerably more than the fibrotic scar, other changes within the lesion could have counteracted some of the expected benefit from reduced fibrosis and increased axonal growth [25].

Examining axonal growth within an inhibitory scar environment can help reaffirm the cross-talk of ECM components throughout the lesion microenvironment, reinforcing the importance of not viewing individual molecules as solely permissive or inhibitory. Jones et al. demonstrated this by introducing fibroblast grafts into rat spinal cord lesions, where NG2-rich cellular substrates later developed throughout the graft [26]. The grafts filled the lesion site and provided a cellular bridge that injured axons could potentially enter and grow through. NG2 colocalized with Schwann-cell markers and endothelial structures within the grafts, and dorsal-column sensory, rubrospinal, and nociceptive axons were still able to enter the grafts and were frequently found extending along these NG2-rich surfaces. The same NG2^+^ surfaces also contained L1 cell adhesion molecule (L1) and laminin, which provide more supportive substrates for axonal growth. In other words, the axons were not interacting with NG2 alone, and their ability to extend through these regions does not mean NG2 itself became permissive. Furthermore, some fibroblast grafts were engineered to secrete nerve growth factor (NGF), which resulted in greater Schwann-cell infiltration and increased axonal growth within the grafts. Simultaneously, NG2 was also greater in the NGF grafts, which is likely attributable to the increase in Schwann cells. Therefore, a strong permissive signal can increase growth without removing inhibitory CSPGs, as their presence alone does not determine whether regeneration occurs; rather, local growth depends on a balance between inhibitory cues and growth-supportive molecules/cells.

Fibronectin provides another supportive example of a major fibrotic ECM component where its presence alone does not necessarily prevent axonal growth. In a rat SCI model, King et al. implanted oriented fibronectin matrices into the lesion [27]. They found substantial axonal growth into these matrices, specifically following the direction and orientation of the fibronectin matrix. Schwann cells infiltrated the implants and surrounded many of the growing axons, with some fibers also becoming myelinated. The combination of these findings shows that fibronectin can exist in an environment that supports axonal extension, but the presence of the Schwann cells limits attributing this growth to fibronectin alone. This same relationship was also seen in human postmortem traumatic SCI tissue. Buss et al. found fibronectin, laminin, and type IV collagen concentrated in Schwann-cell-rich regions where peripheral axonal growth was also present [28]. Therefore, fibronectin itself does not seem to make the environment inhibitory, as the surrounding cellular and ECM makeup can change how axons respond to that region.

Now, the following question arises: if CSPGs are not absolute barriers, how do they still directly inhibit axons? While Jones et al. show that axons can still grow in CSPG-rich environments when permissive cues are strong enough, Shen et al. address the real, receptor-mediated inhibitory effect that CSPGs have on neurons [17,26]. Specifically, they identified PTPσ as a high-affinity neuronal receptor for CSPGs, with binding characterization using neurocan showing that much of the interaction depended on the chondroitin sulfate GAG chains rather than the protein core alone. Inhibition was studied both in vitro and in vivo, allowing the authors to determine whether this receptor-mediated inhibition also affected axonal growth after SCI. The in vitro experiment utilized sensory neurons lacking PTPσ and found they were less inhibited by CSPGs in culture. However, a notable nuance was that the inhibition was reduced but not eliminated, suggesting that PTPσ is only one of the receptors or mechanisms contributing to inhibition of neuronal regrowth through the CSPG-dense ECM. Furthermore, in vivo studies using a dorsal-column SCI crush model demonstrated PTPσ-deficient sensory axons extending farther into CSPG-rich tissue surrounding the lesion than wild-type axons. They still, however, did not show strong regeneration through and beyond the lesion core, further supporting the presence of additional inhibitory mechanisms within the scar environment. Additionally, because CSPGs are distributed across both fibrotic and glial regions of the scar, PTPσ-mediated inhibition is not restricted specifically to fibroblast-derived ECM.

**Figure 3:**
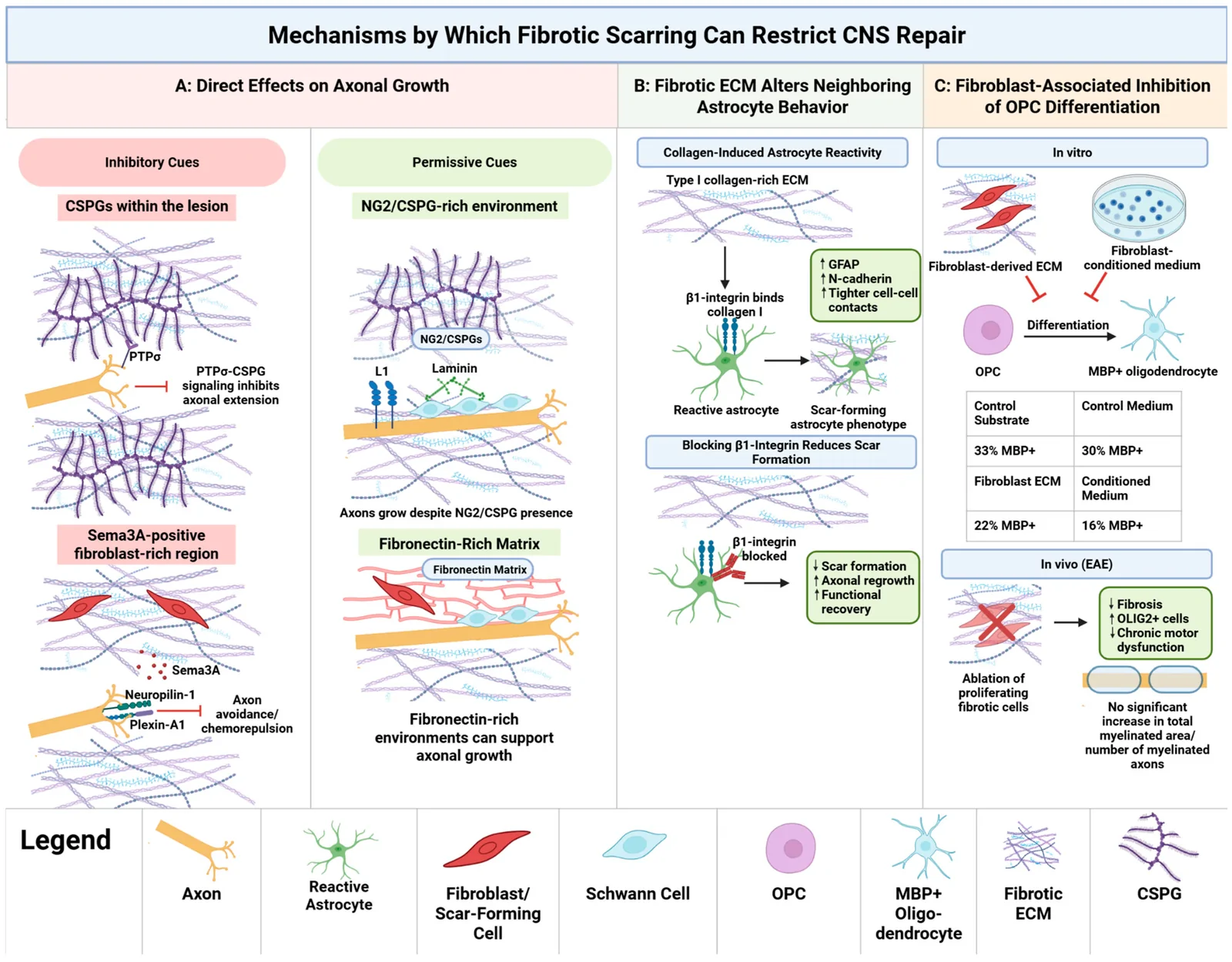
Mechanisms by which fibrotic scarring can restrict central nervous system (CNS) repair. Beyond its initial reparative functions, fibrotic scarring can impair long-term recovery through a variety of mechanisms, including but not limited to direct effects on axonal growth, indirect effects on neighboring astrocytes, and inhibition of oligodendrocyte-lineage repair. (**A**) Across the injury site, different components and factors can provide permissive, inhibitory, or context-dependent signaling cues for neural regeneration. Their interplay is just as important, meaning that the presence of conventional inhibitory ECM molecules does not make the region uniformly nonpermissive. One such inhibitory mechanism comes from direct CSPG signaling, as neurons express the protein tyrosine phosphatase sigma (PTPσ) receptor, which binds the glycosaminoglycan (GAG) chains present on CSPGs and inhibits extension through the CSPG-rich region. Furthermore, sensory neurons lacking PTPσ were less inhibited by CSPGs in vitro, as well as in vivo, where axons extended farther into CSPG-rich tissue surrounding the injury site in a dorsal-column crush model. However, the axons did not extend completely across the region, demonstrating that PTPσ is one inhibitory factor within a larger nonpermissive environment. Another inhibitory factor is semaphorin 3A (Sema3A), which showed strong expression within the fibrotic scar environment following dorsal-column injury. The injured dorsal root ganglion neurons continued to express the receptor components neuropilin-1 and plexin-A1, which together form a receptor complex that allows the axons to respond to Sema3A signaling. In contrast, axonal growth can still occur in neuron-glial antigen 2 (NG2)/chondroitin sulfate proteoglycan 4 (CSPG4)-rich fibrotic environments containing L1 cell adhesion molecule (L1), laminin, and Schwann cells, as well as in fibronectin-rich environments, supporting a dependence on the local balance of inhibitory and permissive cues. (**B**) Type I collagen, which becomes abundant within the fibrotic ECM, interacts with reactive astrocytes through β1-containing integrins and promotes a scar-forming astrocytic phenotype, as seen through increased glial fibrillary acidic protein (GFAP), N-cadherin, and tighter cell–cell contacts. Blocking β1-integrins reduces astrocytic scar formation while increasing axonal regrowth and functional recovery. (**C**) In vitro cell cultures show that fibroblast-derived ECM and fibroblast-conditioned medium reduce oligodendrocyte precursor cell (OPC) differentiation into myelin basic protein (MBP)-positive oligodendrocytes. More specifically, MBP^+^ differentiation dropped from about 33% on control substrate to 22% on fibroblast-derived ECM, while a similar decrease was seen from about 30% in control medium to 16% in fibroblast-conditioned medium. In experimental autoimmune encephalomyelitis (EAE), ablation of proliferating fibrotic cells reduces fibrosis, increases oligodendrocyte transcription factor 2 (OLIG2)-positive oligodendrocyte-lineage cells, and reduces chronic motor disability without significantly increasing total myelinated area or the number of myelinated axons. Therefore, fibrotic scarring can restrict CNS repair through several different cellular and molecular interactions rather than acting only as one uniform physical barrier.

While PTPσ acts as a neuronal receptor mediating CSPG inhibition, semaphorin 3A (Sema3A) provides a different inhibitory cue associated with fibroblast-rich regions of the scar and failed axonal extension. Specifically, Sema3A is a guidance molecule expressed in fibroblast-rich regions of the scar and is associated with axonal chemorepulsion [29]. Pasterkamp et al. found strong Sema3A expression in fibroblast-rich scar tissue, while the injured dorsal root ganglion neurons continued to express neuropilin-1 and plexin-A1, the receptor components needed to respond to Sema3A. Peripheral conditioning was done through a sciatic nerve lesion to increase neuronal intrinsic regenerative capacity before examining their response to the dorsal-column injury. The proposed question with this was whether putting a neuron into a stronger growth state could allow it to cross the scar, and the findings pointed towards a multi-faceted result. The conditioned axons grew much farther toward the spinal lesion but still failed to penetrate Sema3A^+^ scar regions. Importantly, they had extended through some regions containing tenascin-C and CSPGs, suggesting Sema3A-rich regions remained particularly difficult to cross even after increasing intrinsic growth potential. Therefore, increasing a neuron’s regenerative capacity is not always enough to overcome every inhibitory signal in the scar, as the avoidance of Sema3A^+^ fibroblast-rich tissue is consistent with a chemorepulsive effect (Figure 3A).

Moving away from the effect of direct signaling inhibitors on axons, Hara et al. tested whether fibrotic ECM could indirectly influence regeneration by changing the behavior of the surrounding astrocytes forming the scar compartment [30]. In a mouse contusion SCI model, they found type I collagen becoming highly expressed during the scar-forming phase. At 14 days post-injury, scar-forming astrocytes had become associated with the type I collagen-rich regions, whereas reactive astrocytes in type I collagen-poor regions retained a less scar-forming phenotype. Characterization of these ECM organizational findings begins with the fundamental in vitro setup, wherein reactive astrocytes were cultured on collagen I-coated dishes and showed the following changes: increased GFAP, formation of tighter cell–cell contacts, increased N-cadherin, and a shift towards a scar-forming astrocyte phenotype. Mechanistically, the astrocytes interact with collagen through collagen-binding integrins containing β1. Upon blocking these integrins, the collagen-induced transition was reduced, with N-cadherin interference also reducing this shift and supporting β1-integrin and N-cadherin involvement in the transition toward a scar-forming astrocyte phenotype. In vivo, Hara et al. blocked the reactive astrocyte-collagen I interaction following SCI through administration of an anti-β1-integrin antibody, which reduced astrocytic scar formation and increased the presence of growth-associated protein 43-positive (GAP43+) axons at the lesion epicenter and caudal to the injury [30]. Similarly, greater numbers of serotonin-positive and tyrosine hydroxylase-positive fibers were observed following treatment, with many of these fibers also expressing GAP43. Despite these increases, the staining used to identify the axons did not distinguish between regeneration of previously injured fibers and additional growth from axons spared by the injury. Additionally, although locomotor recovery improved following treatment, the study did not directly establish whether these additional axons formed functional connections or were responsible for the improvements in recovery. However, the in vivo effect cannot be attributed specifically to collagen I alone, since β1-containing integrins can interact with multiple extracellular ligands. When considered alongside the culture findings, the results show that fibrotic ECM can influence regeneration indirectly by altering the neighboring astrocytic scar rather than only acting directly on injured axons (Figure 3B).

Outside of axonal growth and astrocytes, fibrosis can also interfere with oligodendrocyte repair, extending its detrimental effects into inflammatory demyelinating disease. In experimental autoimmune encephalomyelitis (EAE), inflammatory demyelinating lesions develop in the spinal cord alongside a fibroblastic scarring response. Yahn et al. mechanistically demonstrated this interplay, as they observed perivascular fibroblasts becoming activated and entering demyelinated spinal cord lesions [31]. These fibroblasts were associated with ECM deposition within demyelinated lesions, and their effects on oligodendrocyte progenitor cell (OPC) differentiation were then isolated experimentally in culture. To observe these effects, purified OPCs were cultured on decellularized fibroblast-derived ECM, and the resulting effects were described. Namely, the percentage of OPCs differentiating into MBP^+^ oligodendrocytes dropped from about 33% on control substrate to 22% on fibroblast ECM, suggesting that fibroblast-deposited matrix itself could interfere with OPC maturation. For a second experiment, OPCs were cultured in fibroblast-conditioned medium, and their differentiation dropped from around 30% to 16%. This provided support that the fibroblast response can affect oligodendrocyte maturation even without requiring direct contact between fibroblasts and OPCs, although the specific soluble products remain unknown. The in vivo effects of fibroblasts on OPCs were later examined by Dorrier et al. [32]. They found collagen I^+^ fibrotic scars developing in inflammatory lesions and persisting into chronic EAE, with lineage tracing and scRNA-seq identifying proliferating CNS fibroblasts as a major source. By selectively ablating proliferating fibrotic cells, overall parenchymal fibrosis was reduced. Following ablation, the animals had less chronic motor disability and more oligodendrocyte transcription factor 2 (OLIG2)-positive oligodendrocyte-lineage cells within lesions. However, there were no significant increases in total myelinated area or the number of myelinated axons, and extensive axonal loss remained, showing that the overall reduction in the fibrotic-cell response improved disease severity and oligodendrocyte-lineage cell numbers, but the measured remyelination outcomes did not significantly improve (Figure 3C).

Collectively, the lesion consists of a fibrotic scar, an astrocytic scar, and an intervening glial-fibrotic interface following SCI. Each compartment plays a distinct role in tissue protection, regeneration, and the development of chronic pathological changes (Table 2).

## 4. Cellular and Molecular Mechanisms Regulating Fibrotic Scar Formation

SCI-induced fibrotic scar formation comes from inflammatory, vascular, and tissue remodeling responses that attempt to stabilize the lesion through ECM deposition. Specific signals regulate the scar-forming fibroblast and perivascular populations as they expand and migrate to the injury site, upregulating ECM production through local profibrotic cues. Their behavior is further shaped by immune-cell signaling, neighboring scar populations, and mechanical cues from the ECM. Overall, the next section will examine how those processes initiate, organize, and maintain the fibrotic scar both in SCI and other disease models.

### 4.1. Inflammatory Cells Promote Fibroblast Recruitment and Profibrotic Signaling

**Figure 4:**
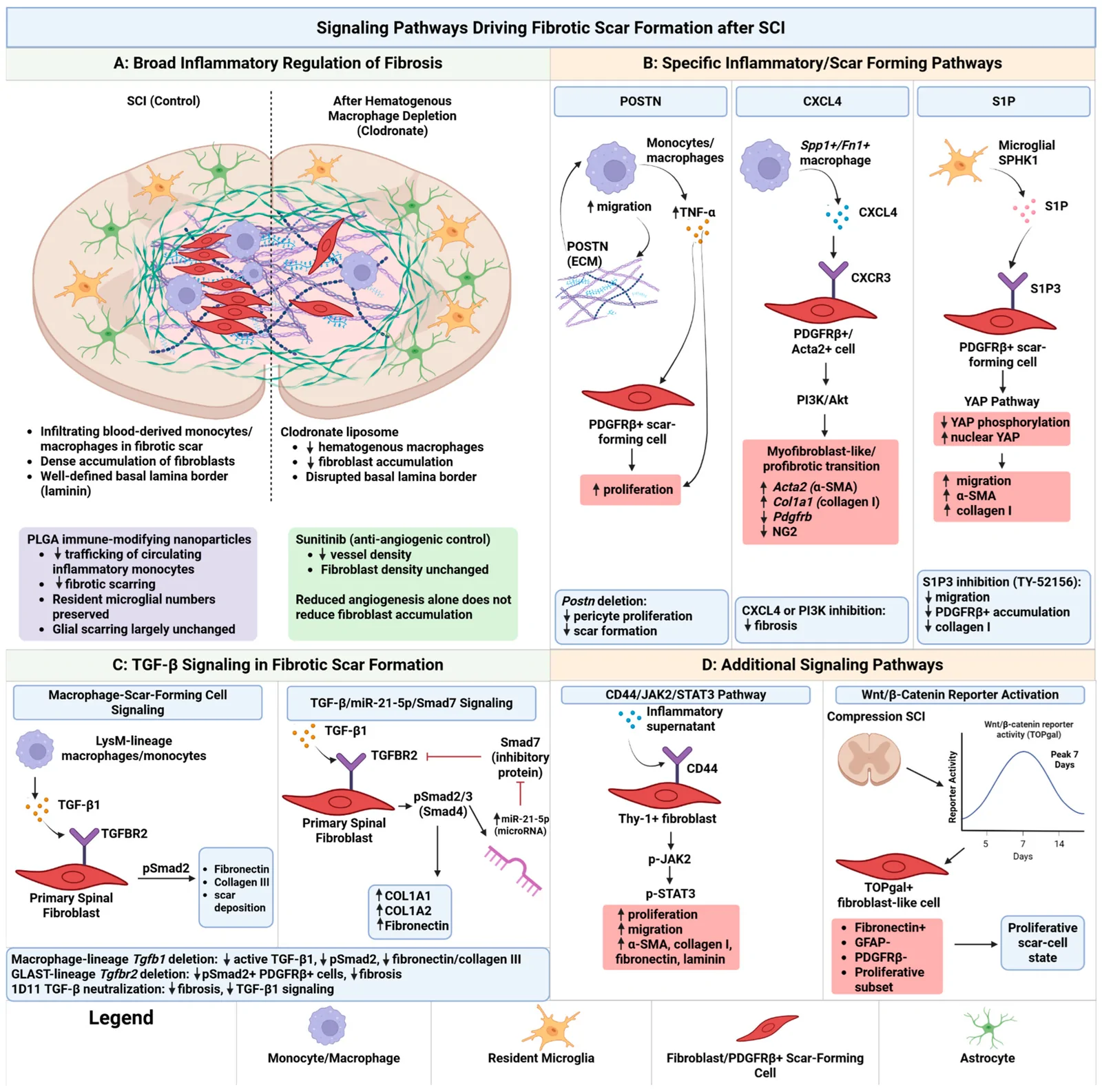
Signaling pathways driving fibrotic scar formation after SCI. Following SCI, broad inflammatory regulation of fibrotic scar formation is coupled with specific immune-to-scar-forming-cell signaling pathways. (**A**) In control SCI, hematogenous monocytes/macrophages and fibroblasts accumulate within the fibrotic scar, with a surrounding laminin^+^ basal-lamina border. Treatment with clodronate liposomes reduces hematogenous macrophage accumulation, fibroblast density, and organization of the basal-lamina border. Sunitinib acts as an anti-angiogenic control to clodronate, as it reduces vessel density without significantly reducing fibroblast accumulation and shows that the clodronate-associated reduction in fibroblast populations is not simply attributable to decreased angiogenesis. Separately, poly(lactide-co-glycolide) (PLGA) immune-modifying nanoparticles reduce trafficking of circulating inflammatory monocytes into the lesion, decreasing fibrosis while comparatively preserving resident microglial populations and glial scarring. (**B**) Periostin (POSTN) increases within the ECM of the developing fibrotic scar and is primarily associated with platelet-derived growth factor receptor beta-positive (PDGFRβ^+^) scar-forming cells. Recombinant POSTN increases monocyte/macrophage migration and tumor necrosis factor alpha (TNF-α) expression by monocytes/macrophages. TNF-α then increases proliferation of the PDGFRβ^+^ population, whereas *Postn* deletion reduces proliferation of this cell population and overall scar formation. C-X-C motif chemokine ligand 4 (CXCL4) is produced by *Spp1^+^/Fn1^+^* macrophages and acts through C-X-C motif chemokine receptor 3 (CXCR3) on PDGFRβ^+^/*Acta2^+^* cells that the authors classified as pericytes undergoing pericyte-to-myofibroblast transition. CXCL4/CXCR3 signaling activates phosphoinositide 3-kinase/protein kinase B (PI3K/Akt) signaling, leading to increases in *Acta2*/alpha-smooth muscle actin (α-SMA) and *Col1a1*/collagen I and decreases in *Pdgfrb* and NG2/CSPG4, shifting the population toward a more myofibroblast-like, profibrotic phenotype. CXCL4 neutralization or PI3K inhibition reduces the fibrotic response. Following SCI, sphingosine kinase 1 (SPHK1), an enzyme that produces sphingosine-1-phosphate (S1P), increases in ionized calcium-binding adaptor molecule 1-positive (Iba1^+^) cells, while spinal-cord S1P levels also rise. S1P acts through S1P receptor 3 (S1P3) on PDGFRβ^+^ scar-forming cells, promoting migration while also decreasing phosphorylation of Yes-associated protein (YAP) and increasing its nuclear localization. YAP activation is associated with increased α-SMA and collagen I, while inhibition of S1P3 with TY-52156 reduces migration, PDGFRβ^+^ cell accumulation, and collagen I deposition. (**C**) Macrophages provide an endogenous source of transforming growth factor beta 1 (TGF-β1), which acts on scar-forming cells through TGFBR2/Smad signaling. Lysozyme M (LysM)-lineage macrophages and monocytes provide TGF-β1, which acts through TGFBR2 on GLAST-lineage/PDGFRβ^+^ scar-forming cells, increasing phosphorylated Smad2 (pSmad2) and promoting fibronectin and collagen III scar deposition. Perturbing components of this signaling pathway reduces both fibrotic ECM deposition and pathway activation, including macrophage-lineage *Tgfb1* deletion, GLAST-lineage *Tgfbr2* deletion, and transforming growth factor beta (TGF-β) neutralization with 1D11. Intracellularly, TGF-β1 treatment of primary spinal fibroblasts increases *COL1A1*, *COL1A2*, fibronectin, and microRNA-21-5p (miR-21-5p), while decreasing Smad7. miR-21-5p suppresses Smad7 expression, and because Smad7 normally inhibits TGF-β signaling, its reduction reinforces the fibrotic response. Manipulating miR-21-5p supports this relationship, as overexpression enhances fibrosis-related gene expression, whereas inhibition decreases these genes and increases Smad7. (**D**) Exposure of thymocyte differentiation antigen 1-positive (Thy-1^+^) fibroblasts to an inflammatory conditioned medium activates the cluster of differentiation 44 (CD44)/Janus kinase 2 (JAK2)/STAT3 pathway. Increased phosphorylation of JAK2 and STAT3 is associated with greater fibroblast proliferation and migration and increased α-SMA, collagen I, fibronectin, and laminin expression, whereas inhibition of CD44/JAK2/STAT3 reduces these responses. Canonical Wnt/β-catenin signaling was examined following compression SCI using the T-cell factor/lymphoid enhancer factor (TCF/LEF) optimal promoter/β-galactosidase (TOPgal) reporter. TOPgal becomes active when canonical Wnt signaling stabilizes β-catenin, allowing β-catenin to enter the nucleus and promote TCF/LEF-dependent transcription. Reporter activity first appeared around day 5, peaked around day 7, and declined by day 14. TOPgal^+^ cells were fibronectin^+^, GFAP^−^, PDGFRβ^−^ fibroblast-like cells, with a subset undergoing proliferation, associating canonical Wnt/β-catenin activity with the proliferative scar-cell state.

Following traumatic SCI, blood-derived macrophages accumulate in the lesion compartment alongside scar-forming fibroblasts. To examine whether macrophages are merely present alongside fibrosis or play a role in driving the process, Zhu et al. examined a mouse contusion SCI model (described in the Detrimental Effects section) and observed that hematogenous macrophages became concentrated in the fibrotic core while resident microglia were more associated with the surrounding astrocytic, glial-scar region [25]. Reduction in these hematogenous macrophages with clodronate reduced fibroblast density at both 7 and 14 days, with the dense peripheral fibroblast population and associated basal-lamina formation also being reduced. The control sunitinib reduced vascular density but did not significantly reduce fibroblast accumulation, indicating that the reduced fibroblast density after macrophage depletion was not explained simply by reduced angiogenesis and that macrophages promote fibroblast recruitment or accumulation after SCI. Independent modulation of circulating monocytes supported the same general relationship [39]. Using poly(lactide-co-glycolide) immune-modifying nanoparticles, Jeong et al. reduced trafficking of circulating inflammatory monocytes into the injured spinal cord, which was accompanied by decreased fibrotic scarring. Resident microglial numbers were comparatively preserved, while glial scarring was also largely unaffected. Overall, this strengthens the argument that infiltrating blood-derived immune cells are upstream regulators of the fibrotic response rather than simply cells that accumulate after scar formation, opening up the possibility of understanding the biochemical mechanisms underlying these proliferative and structural changes (Figure 4A).

While previous studies showed the broad importance of inflammatory signaling on fibrotic scar formation, more specific experiments have since identified individual molecular pathways through which these cells can affect scar-forming populations. Yokota et al. characterized the role of the ECM protein periostin (POSTN) in the proliferation of scar-forming cells [40]. Following mouse T9 contusive SCI, POSTN expression increased and peaked at approximately 7 days post-injury, corresponding temporally to active fibrotic scar formation. More specifically, POSTN was predominantly associated with PDGFRβ^+^ cells, which Yokota et al. termed “scar-forming pericytes.” By genetically deleting the *Postn* gene, *Postn*-null mice exhibited decreased proliferation of PDGFRβ^+^ scar-forming cells and overall reduced scar formation. Based on this knockout, POSTN was shown to contribute to the fibrotic response rather than simply being present within the scar. The connection between POSTN and PDGFRβ^+^ cell proliferation becomes clearer when considering the following findings: recombinant POSTN increased monocyte/macrophage migration in vitro, *Postn*-deficient mice had reduced inflammatory-cell infiltration, recombinant POSTN increased tumor necrosis factor alpha (TNF-α) expression by monocytes/macrophages, and TNF-α expression was lower after *Postn* deletion. Separately, the authors isolated cells they classified as spinal-cord pericytes and exposed them to recombinant TNF-α, which increased their proliferation in a concentration-dependent manner. Therefore, the proposed interaction involves POSTN in the developing scar attracting monocytes and macrophages and increasing macrophage TNF-α expression, which then promotes proliferation of PDGFRβ^+^ scar-forming cells.

Building on this, Li et al. identified an even more specific macrophage-derived signaling pathway in T10 complete crush SCI mice by studying C-X-C motif chemokine ligand 4 (CXCL4) after injury [41]. scRNA-seq analysis identified an *Spp1^+^*/*Fn1^+^* macrophage subset with strong profibrotic and ECM-regulatory characteristics, and *Cxcl4* expression was particularly enriched in this population. Flow cytometry and immunostaining further supported these macrophages as an important source of CXCL4 at the injury site after SCI. To study its effect, they examined a PDGFRβ^+^/*Acta2^+^* cell population that they classified as pericytes undergoing a pericyte-to-myofibroblast transition and showed that these cells expressed C-X-C motif chemokine receptor 3 (CXCR3), the receptor examined for CXCL4 signaling. Treatment with exogenous CXCL4 increased *Acta2* and *Col1a1* while decreasing *Pdgfrb* and NG2 in a concentration-dependent manner, supporting a role for CXCL4 in shifting the population toward a more myofibroblast-like/profibrotic phenotype. Furthermore, they isolated *Spp1^+^*/*Fn1^+^* macrophages and cocultured them with the responding PDGFRβ^+^/*Acta2^+^* cells, resulting in an increase in *Acta2*/*Col1a1* and a decrease in *Pdgfrb*/NG2. These changes were reduced by knocking down *Cxcl4* in the macrophages or *Cxcr3* in the responding cells, providing strong evidence that the macrophage effect was mediated through the CXCL4/CXCR3 axis. To identify the downstream mechanism, Li et al. next analyzed CXCL4-treated cells and found enrichment of phosphoinositide 3-kinase/protein kinase B (PI3K/Akt) signaling, with CXCL4 exposure also increasing PI3K phosphorylation [41]. Inhibition of PI3K with LY294002 or Akt with MK2206 reduced the CXCL4-associated increase in alpha-smooth muscle actin (α-SMA) and collagen I while restoring PDGFRβ and NG2 expression, supporting PI3K/Akt as a downstream mediator of the CXCL4 response. The pathway was also supported in vivo, where CXCL4 neutralization or PI3K inhibition reduced the fibrotic response following SCI.

Additionally, Yu et al. demonstrated that macrophages are not the only inflammatory population capable of directly signaling to PDGFRβ^+^ scar-forming cells following SCI [42]. They specifically showed that microglia could influence this population through sphingosine-1-phosphate (S1P) signaling. In the T9 crush model, sphingosine kinase 1 (SPHK1), the enzyme that produces S1P, increased in ionized calcium-binding adaptor molecule 1-positive (Iba1^+^) cells after injury. Along with this enzyme, its product S1P also increased in the spinal cord and reached its peak around 3 days post-injury. After depleting microglia with PLX5622, this increase in S1P was not as substantial, suggesting that microglia play an important role in S1P levels following SCI. Following this signal, they observed PDGFRβ^+^ cells accumulating in the injury site, most of which expressed sphingosine-1-phosphate receptor 3 (S1P3). Upon binding, they saw that S1P exposure as well as conditioned medium from injured-model microglia increased migration of cultured pericytes, followed by an observed reduction in migration when S1P3 was blocked with TY-52156. Both experiments strengthened the connection between microglia-derived S1P and PDGFRβ^+^ cell migration, since blocking S1P3 reduced the migratory response. Beyond migration, Yu et al. also found that S1P signaling increased α-SMA and collagen I, suggesting that the responding cells were also shifting toward a more fibrotic state [42]. The authors next looked inside the cell at Yes-associated protein (YAP), a transcription factor regulating fibrotic gene expression, to determine what was happening downstream of S1P3. After S1P/S1P3 signaling, YAP phosphorylation decreased, and more YAP was found localized in the nucleus. They then used knockdown experiments to test whether S1P3 and YAP were actually part of the same pathway. Knockdown of S1P3 reduced YAP activation and reduced α-SMA and collagen I, while knockdown of YAP reduced the S1P-induced increase in the fibrotic markers, placing YAP downstream of S1P3 in the fibrotic deposition mechanism. Treating SCI mice with the S1P3 inhibitor TY-52156 reaffirmed these results in vivo, as it led to reduced PDGFRβ^+^ cell accumulation and collagen I deposition later after injury. Therefore, Yu provides another immune-to-scar-cell pathway, where microglia-associated S1P signaling through S1P3/YAP promotes migration and fibrotic activation of PDGFRβ^+^ cells (Figure 4B).

### 4.2. TGF-β Signaling Promotes Fibroblast Activation and Fibrotic ECM Deposition

Transforming growth factor beta (TGF-β) plays a significant role in the fibrotic response, as seen throughout various biochemical models. One foundational experiment conducted by Logan et al. examined the injured rat brain and found that TGF-β1 becomes elevated at the injury site [43]. Furthermore, they found that injecting exogenous TGF-β1 or giving a neutralizing TGF-β1 antibody yielded respective increases and decreases in the scarring response. This causal relationship was further explored by Pan et al. using an adult mouse T10 crush SCI model [44]. Rather than only examining whether TGF-β was present after injury, Pan et al. first reduced LysM-lineage macrophage/monocyte cells and found decreases in both fibrotic scar formation and active TGF-β1, along with reduced phosphorylation of Smad2, a downstream marker of TGF-β signaling. They then more specifically deleted *Tgfb1* from the macrophage/monocyte lineage, which again reduced fibronectin- and collagen III^+^ scarring while also decreasing TGF-β1 levels and phosphorylated Smad2 (pSmad2) signaling. The connection between macrophage-derived TGF-β1 and scar-forming cells was further supported by the presence of pSmad2 in PDGFRβ^+^ cells after SCI, with this population decreasing when macrophage-lineage *Tgfb1* was deleted. These findings therefore move beyond the earlier observation that TGF-β can promote CNS scarring and identify macrophage-derived TGF-β1 as one source contributing to fibrotic scar formation after SCI.

Having established macrophage-derived TGF-β1 as a source of profibrotic signaling after SCI, Pan et al. next tested whether scar-forming cell populations needed to receive the signal for the ECM components to be deposited [44]. Using Glast-CreERT2 mice, which use a second-generation tamoxifen-inducible Cre-estrogen receptor system, they deleted *Tgfbr2* in GLAST-lineage cells (referred to by the authors as pericytes), which resulted in less fibronectin, collagen III, pSmad2, and fewer overall pSmad2^+^/PDGFRβ^+^ cells. Since those cells were receiving less TGF-β signaling and induced less fibrosis, TGF-β signaling in that GLAST-lineage cell population contributes to scar formation. Afterward, Pan et al. systemically treated SCI mice with 1D11, a TGF-β-neutralizing antibody, which led to a reduction in fibronectin, collagen III, and pSmad2 signaling [44]. Overall, these two separate methods of blocking TGF-β signaling yielded the same general result, meaning TGF-β is functionally important for fibrosis after SCI.

Inside the fibroblast, TGF-β1 facilitates changes to the transcription of scar-related genes. Wang et al. studied transcriptional patterns in primary spinal fibroblasts after addition of TGF-β1 [45]. Upon addition, the fibroblasts increased *COL1A1*, *COL1A2*, and fibronectin in a dose-dependent manner, indicating that TGF-β1 directly pushes spinal fibroblasts toward a more fibrotic state, resulting in greater ECM production. Additional targets modulated by TGF-β1 introduction included miR-21-5p, a microRNA that increased after treatment, and Smad7, an inhibitory signaling protein whose expression decreased. Conversely, overexpressing miR-21-5p resulted in an increase in fibrotic gene expression, while inhibiting miR-21-5p decreased fibrotic gene expression but increased Smad7. These findings supported miR-21-5p as a negative regulator of Smad7 expression, while Smad7 itself acts as a negative regulator of TGF-β signaling, creating a double-negative regulatory mechanism that amplifies TGF-β signaling and the resulting fibrotic response (Figure 4C).

### 4.3. CD44/JAK2/STAT3 and Wnt/β-Catenin Signaling Regulate Scar-Forming Cells

Outside of TGF-β, other intracellular pathways have also been implicated in regulating fibroblast behavior after SCI. Guo et al. examined cluster of differentiation 44 (CD44), a cell-surface receptor that became upregulated during fibrotic scar formation and was primarily associated with thymocyte differentiation antigen 1-positive (Thy-1^+^) fibroblasts [46]. Blocking CD44 with the monoclonal antibody IM7 decreased collagen I, fibronectin, laminin, and α-SMA after injury, with reductions in the fibrotic response still being observed at chronic time points. The effect was studied more directly in vitro by exposing fibroblasts to inflammatory supernatant, which increased their proliferation and migration along with expression of fibrosis-associated proteins. Addition of IM7 reduced each of these responses, indicating that CD44 helps fibroblasts respond to signals present in the inflammatory environment. Looking further downstream, inflammatory stimulation increased phosphorylation of Janus kinase 2 (JAK2) and STAT3, whereas CD44 inhibition reduced the activation of both proteins. Direct inhibition of JAK2/STAT3 similarly decreased fibroblast proliferation and migration while reducing α-SMA, collagen I, fibronectin, and laminin expression. However, inhibition of this pathway did not significantly change pSmad2, suggesting that CD44/JAK2/STAT3 can regulate the fibrotic response without simply acting through the TGF-β/Smad mechanism described in the previous section.

Yamagami et al. examined canonical Wnt/β-catenin signaling over time following compression SCI using a reporter system that produces a detectable signal when the pathway becomes active [47]. In this pathway, Wnt signaling stabilizes the β-catenin protein, allowing it to enter the nucleus and regulate transcription, so reporter^+^ cells indicate cells where Wnt/β-catenin signaling is active. By monitoring throughout the scar-forming phase, they saw reporter activity beginning around day 5 and rising until it peaked at day 7, declining thereafter until day 14. The reporter^+^ cells in the injury site were mostly fibronectin^+^, GFAP-negative, fibroblast-like, and highly proliferative, showing that Wnt/β-catenin signaling was active specifically during the period when scar-forming fibroblast-like cells were expanding. This demonstrated a non-causal association between the Wnt pathway and fibroblast proliferation, as its activity coincided with scar formation without the pathway itself being experimentally blocked (Figure 4D).

### 4.4. EphB2/Ephrin-B2 Signaling Organizes the Fibrotic-Glial Scar Boundary

**Figure 5:**
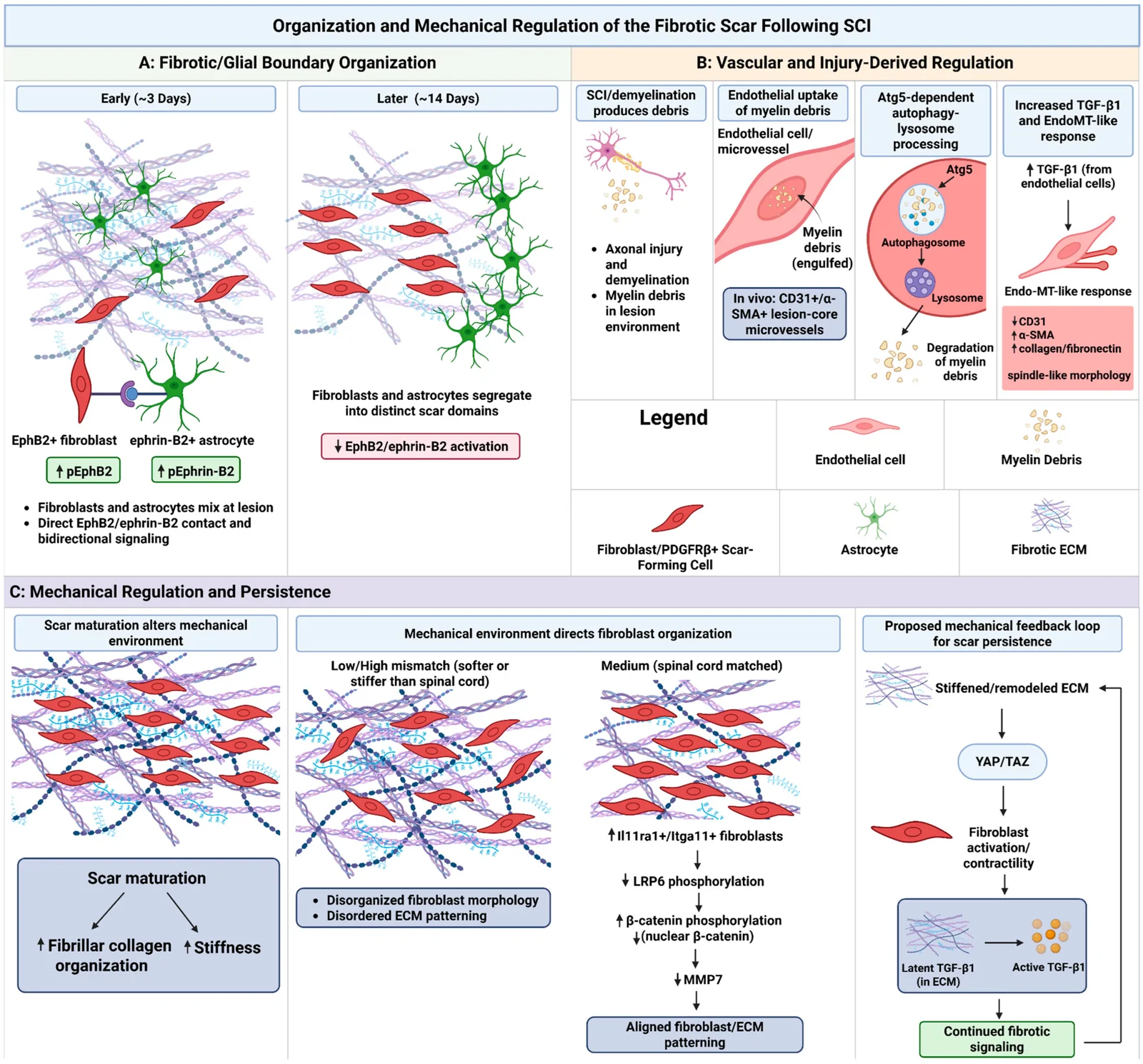
Organization and mechanical regulation of the fibrotic scar following SCI. Formation of the fibrotic scar is regulated by factors including fibroblast–astrocyte boundary organization, injury-derived vascular responses, and mechanical properties of the maturing fibrotic ECM. (**A**) In early stages of scar formation, fibroblasts and reactive astrocytes are initially intermixed following SCI. Their distribution becomes more organized through interactions between ephrin type-B receptor 2 (EphB2) and ephrin-B2, present on meningeal fibroblasts and reactive astrocytes, respectively. This requires cell–cell contact since both are membrane-bound proteins. After the injury, EphB2/ephrin-B2 phosphorylation increases, supporting bidirectional signaling while the cells continue to interact. Throughout development, EphB2/ephrin-B2 activation decreases, indicating that the interaction between the cells occurs mostly during border formation. (**B**) Demyelination following SCI leaves myelin debris in the injury site, which is engulfed by microvascular endothelial cells. Inside these cells, the debris is processed through the autophagy-lysosome pathway, resulting in increased expression of TGF-β1, α-SMA, collagen, and fibronectin, decreased platelet endothelial cell adhesion molecule-1 (CD31) expression, and development of a more spindle-like cell morphology. This response can be described as endothelial-to-mesenchymal transition (EndoMT)-like, with TGF-β neutralization reducing the myelin-induced changes. Additionally, autophagy-related 5 (*Atg5*)-deficient endothelial cells do not show the same α-SMA increase or spindle-like response after myelin exposure, supporting the role of processing engulfed debris in the profibrotic response. Therefore, vascular cells can contribute to the profibrotic lesion response after processing injury-derived myelin debris. (**C**) As the scar matures, fibrillar collagen organization and stiffness increase. In rat T10 transection SCI, hydrogels composed of hyaluronic acid-graft-dopamine (HADA) and the designer peptide HGF-(RADA)4-DGDRGDS (HRR) with Low, Medium, and High mechanical strengths were used to examine the effect of the mechanical environment on scar organization. The Medium condition most closely matched native spinal cord mechanics and produced a more longitudinally aligned organization of collagen I, collagen IV, fibronectin, and laminin, whereas the Low and High conditions produced denser and more disorganized ECM. PDGFRβ^+^/*Col1a1^+^* fibroblasts remained present in all conditions, indicating that the mechanical environment changed fibroblast behavior and ECM organization rather than simply reducing or eliminating the scar-forming population. Specifically, within the medium condition, single-cell RNA sequencing (scRNA-seq) found increased abundance of an interleukin 11 receptor subunit alpha 1-positive/integrin subunit alpha 11-positive (*Il11ra1^+^/Itga11^+^*) fibroblast subset associated with ECM assembly and migration and aligned with the longitudinal matrix. This condition was also associated with decreased low-density lipoprotein receptor-related protein 6 (LRP6) phosphorylation, nuclear β-catenin, and matrix metalloproteinase 7 (MMP7), along with increased β-catenin phosphorylation, supporting an LRP6/Wnt/β-catenin/MMP7-associated pathway connecting the mechanical environment to fibroblast migration and ECM organization. A proposed explanation based on non-SCI models for mechanical stiffness introduces a feedback loop, wherein stiffened ECM from the scar environment increases mechanosensitive YAP and transcriptional coactivator with PDZ-binding motif (TAZ) activity. These transcriptional cofactors increase fibroblast activation, contractility, and profibrotic gene expression. Myofibroblast contraction can mechanically activate latent TGF-β1 stored in the ECM, further supporting fibroblast activation. This resulting stiffness can then increase YAP/TAZ signaling, furthering the proposed positive-feedback loop for continued fibrosis. Therefore, fibrotic scar organization can be influenced by surrounding cell populations, injury-derived factors, and changes in the mechanical environment of the scar.

After SCI, fibroblasts move into the injury site while reactive astrocytes accumulate around it, forming an astrocyte-bound glial scar around the fibrotic compartment. Bundesen et al. examined how these two populations became organized into separate regions of the scar by examining a rat thoracic spinal cord transection model [48]. Specifically, they examined the ephrin type-B receptor 2 (EphB2)/ephrin-B2 signaling pair, where EphB2 is expressed by invading meningeal fibroblasts while ephrin-B2 is a membrane-bound ligand expressed by reactive astrocytes. Since ephrin-B2 is membrane-bound, the cells need to be in direct contact for signaling. During the early stages of scar formation, fibroblasts and astrocytes were intermixed, and both proteins had increased phosphorylation indicative of bidirectional signaling. As the scar matured, the two cell populations began to occupy more separate regions, with clear segregation along the scar boundary being prominent around 14 days post-injury. At that same time, EphB2/ephrin-B2 activation declined, meaning the strongest signaling happened before or during the period when the boundary was forming, not after the populations were fully separated. Therefore, the findings support a role for EphB2/ephrin-B2 signaling in the early organization of the fibroblast–astrocyte border, since activation of both proteins was highest during the period when the two populations were still interacting and beginning to separate (Figure 5A).

### 4.5. Endothelial Processing of Myelin Debris Promotes a Profibrotic Response

SCI causes demyelination in the injury site, leaving behind myelin debris. Zhou et al. asked whether microvascular endothelial cells do more than just form blood vessels during repair, and ended up observing that newly formed microvessels in the lesion core began engulfing myelin debris within days of SCI [49]. Upon engulfing the myelin debris, it was internalized and processed through the autophagy-lysosome pathway. Prolonged myelin-debris exposure changed the behavior of these endothelial cells, apparent through their increased expression of collagen genes and fibronectin. The endothelial cells also began showing features of an endothelial-to-mesenchymal transition (EndoMT)-like state, where they decreased platelet endothelial cell adhesion molecule-1 (CD31), increased α-SMA, and took on fibroblast-like spindle morphology indicative of a profibrotic phenotype [49]. The debris engulfment increased TGF-β1 expression in endothelial cells, and subsequent blocking of TGF-β signaling with a pan-TGF-β neutralizing antibody reduced the myelin-induced EndoMT-like changes. The importance of actually processing the engulfed debris was examined using autophagy-related 5 (*Atg5*), a component of the autophagy machinery. *Atg5*-deficient endothelial cells did not show the same spindle-like morphology or α-SMA increase after myelin exposure, showing that processing of the debris was required following engulfment in order for the fibrotic transition to occur. This was supported in vivo, as lesion-core microvessels after SCI showed colocalization of CD31 and α-SMA, which is likely indicative of an EndoMT-like response occurring (Figure 5B).

### 4.6. Matrix Mechanics Regulate Fibroblast Organization and May Reinforce Chronic Fibrosis

As previously described in the components section, Manesco et al. found that the injured spinal cord both becomes mechanically harder as the scar matures and increases fibrillar collagen organization [12]. This gives rise to the question of whether mechanical properties can feed back onto fibroblasts and alter the architecture of the resulting scar. Xiao et al. directly tested this proposition by implanting hyaluronic acid-graft-dopamine (HADA)/HGF-(RADA)4-DGDRGDS (HRR) hydrogels with different mechanical strengths at the injury site following rat T10 transection SCI [50]. The Medium-strength hydrogel most closely matched the mechanical properties of the wild-type thoracic section of the spinal cord, with the rest of the gels sharing similar overall structure and composition with their only variation being mechanical strength. The other gel types (Low and High strength) resulted in dense and disorganized ECM organization, while the mechanically matched Medium condition produced a much more parallel arrangement aligning longitudinally with the spinal cord. This organization was observed across several ECM components, including collagens I/IV, fibronectin, and laminin, indicating that the mechanical environment changed how the ECM was organized, not simply whether the ECM was present. This coincides with PDGFRβ^+^/*Col1a1*^+^ fibroblasts infiltrating the matrix in all groups, showing a mechanistic change in fibroblast behavior rather than downregulation or elimination. To determine what was changing inside the fibroblasts, Xiao et al. next used scRNA-seq at 2 weeks post-injury and identified a fibroblast subset characterized by *Il11ra1* and *Itga11* that was especially enriched in the Medium group [50]. These cells were associated with ECM assembly and cell migration, and they aligned with the longitudinal pattern of the remodeled matrix. The Medium condition also had lower matrix metalloproteinase 7 (MMP7) expression, so the authors tested whether MMP7 was controlling this fibroblast pattern. Adding MMP7 reduced the *Il11ra1^+^*/*Itga11^+^* population and disrupted the parallel *Col1a1* arrangement, while inhibiting MMP7 increased this fibroblast subset and produced a more aligned matrix even in the SCI, High, and Low conditions. They then traced this response further upstream to low-density lipoprotein receptor-related protein 6 (LRP6) and β-catenin. In the Medium condition, LRP6 phosphorylation was reduced, β-catenin phosphorylation increased, and less β-catenin entered the nucleus, which corresponded with lower MMP7 expression. Manipulating LRP6 phosphorylation reversed these effects, supporting an LRP6/Wnt/β-catenin/MMP7 pathway that allows fibroblasts to respond to the mechanical environment and alter how they migrate and organize the fibrotic ECM.

After the scar itself has formed, the stiffening environment can potentially maintain fibrosis, facilitating chronic injury. It has already been established that the scar becomes mechanically different as it matures and fibroblasts can respond to these differences in mechanical stiffness [12,50]. Therefore, if fibroblasts can sense mechanics, then the scar itself may be able to influence whether they remain activated. Yu et al. previously showed that YAP can regulate fibrotic behavior downstream of S1P3 signaling in PDGFRβ^+^ cells [42]. This makes the related work of Jorgenson et al. especially relevant, as they studied transcriptional coactivator with PDZ-binding motif (TAZ), a closely related transcriptional co-regulator that is also highly mechanosensitive [51]. Foundationally, YAP/TAZ can act as a bridge between mechanical and biochemical signaling, which is especially important considering their role in inducing fibrotic gene expression. When TAZ was activated, increases in fibroblast proliferation, contractility, and profibrotic gene expression were observed, with some of these differences becoming stronger in stiffer matrices. TAZ-dependent collagen expression was also found to increase with TGF-β, further connecting mechanical signaling through YAP/TAZ to the biochemical signaling pathway using TGF-β. This connection was further characterized by Wipff et al., as they showed the contractile force generated by myofibroblasts could mechanically activate latent TGF-β1 stored in the ECM [52]. Their overarching finding indicated that myofibroblast contraction can itself activate latent TGF-β1 already stored in the ECM, allowing TGF-β signaling to increase without requiring a completely new source of the growth factor. Altogether, stiffening of the scar could promote mechanosensitive YAP/TAZ activity that keeps fibroblasts activated and contractile, further activating TGF-β from the ECM for sustained fibrotic signaling. While the individual steps are supported, the entire feedback loop has not been shown in SCI, so it provides a possible mechanism for maintaining chronic fibrosis but is still open to further research (Figure 5C).

In summary, although reparative and pathological fibrosis utilize similar molecular and cellular mechanisms, both processes can be fundamentally distinguished through a coordinated temporal and spatial framework, as they follow drastically different trajectories across cell dynamics, ECM architecture, biomechanics, and molecular signal propagation (Table 3).

## 5. Therapeutic Strategies for Modulating Fibrotic Scarring

As established in previous sections, the fibrotic scar contributes both towards wound repair and later regenerative restriction. Therefore, therapeutic approaches cannot simply aim to eliminate scar formation altogether. This is especially clear from studies manipulating GLAST-lineage scar-forming cells following SCI, where strong suppression of this cell population interfered with wound closure and tissue stability while moderate attenuation reduced fibrosis while preserving structural integrity and improving axonal growth and sensorimotor recovery [7,8]. Based on this foundation, many therapies have approached attenuation from different directions, targeting separate components throughout different stages with the ultimate goal of reducing the excessive and persistent fibrosis that develops after its initial wound-healing functions have been established.

### 5.1. Inhibiting Profibrotic Signaling and Scar-Forming Cell Activity

Several therapeutic approaches have been built from inhibiting stages involved in the previously described TGF-β profibrotic pathway. Logan et al. established this early on in injured rat brains, where they found that neutralizing endogenous TGF-β1 reduced fibrotic scar deposition [43]. Furthermore, Yoshioka et al. inhibited the TGF-β type I receptor kinase in a mouse nigrostriatal pathway transection model with LY-364947, which resulted in reduced fibronectin and collagen IV deposition as well as more tyrosine hydroxylase^+^ dopaminergic axons extending further through the injury site [62]. While these studies establish the TGF-β pathway as pharmacologically targetable, they still require testing in traumatic SCI to support a broader neurological interpretation of the findings. Addressing this, Pan et al. tested similar mechanisms in mouse T10 crush SCI by systemically administering the TGF-β-neutralizing antibody 1D11 three times per week for 56 days [44]. In these animals, they found reduced fibronectin, collagen III, and downstream TGF-β/Smad signaling and increased neural/axonal measures across the lesion (protein gene product 9.5 [PGP9.5], βIII-tubulin, and 5-hydroxytryptamine [5-HT]) along with improved behavioral outcomes (BMS, von Frey, and hot plate). Although these findings showed increases in neuronal and axonal markers following treatment, the immunostaining did not establish whether the additional fibers represented regeneration of previously injured axons or preservation and growth of spared populations. The behavioral improvements also could not be attributed specifically to axonal regeneration, particularly since TGF-β inhibition affected several other components of the injury response. However, 1D11 acts as a systemic neutralizer, affecting many injury processes beyond fibrosis since it is not a fibroblast-specific treatment. The reduction in fibrotic scarring was also accompanied by increased GFAP^+^ glial scarring, further demonstrating that suppressing TGF-β altered the broader lesion response. Therefore, while TGF-β inhibition reduces fibrosis alongside improved recovery, the functional benefits cannot be attributed solely to changes in the fibrotic scar. This makes TGF-β a strongly supported experimental anti-fibrotic target after SCI, although its broad biological activity limits the selectivity of systemic pathway inhibition.

Rather than broadly suppressing TGF-β, some studies have instead targeted receptors associated more directly with scar-forming cells. Guo et al. tested this using intrathecal IM7 to block CD44 [46]. After SCI, CD44 increased mainly in Thy-1^+^ fibroblasts, where it regulated proliferation, migration, and ECM expression through JAK2/STAT3 signaling. The resulting outcomes showed reduced fibrotic scar formation at both 2 and 12 weeks, with specific reductions in collagen I, fibronectin, laminin, and α-SMA. In terms of axonal regeneration, Guo et al. used BDA tracing to examine CST axons following IM7 treatment, finding more labeled fibers extending through the fibrotic lesion compared to untreated SCI animals [46]. The authors also examined whether these fibers could have been spared following the initial injury, as earlier tracing experiments showed no BDA-labeled CST axons within the region caudal to the lesion at 2 weeks post-SCI. These findings supported regeneration of injured CST axons rather than simply greater preservation of existing fibers, although the study did not directly establish whether these axons formed functional connections. Improvements in motor and sensory recovery were also observed following treatment, but their contribution from CST regeneration specifically could not be isolated from the other effects of CD44 inhibition. Although CD44 itself is broadly expressed across various vertebrate cells, Guo et al. found that its expression was primarily associated with Thy-1^+^ fibroblasts in the injured spinal cord, and the lower IM7 dose reduced fibrosis without significantly changing GFAP^+^ astrocytic scarring. Li et al. took a similar approach through inhibition of PDGFRβ signaling in scar-forming fibroblasts with intrathecal delivery of SU16f beginning 3 days post-SCI, resulting in reduced PDGFRβ^+^ fibroblast proliferation, fibronectin, and laminin across the scar [63]. The overall lesion size and chronic inflammatory-cell accumulation also decreased, alongside increases in neurofilament-positive and 5-HT^+^ axons and improvements in locomotor recovery. However, increased staining for these axonal markers alone does not distinguish regeneration from preservation or sprouting of spared fibers, meaning that the relationship between the additional axons and improved recovery could not be directly determined from these findings. Li et al. used PDGFRβ as a marker of the fibrotic scar-forming fibroblasts in their model [63]. More recent lineage-tracing work, however, has shown that PDGFRβ is also expressed by other perivascular populations [5]. Since lesion size and chronic inflammation were also reduced after SU16f treatment, it is difficult to separate the contribution of reduced fibrosis from these other changes. These studies therefore get closer to targeting the scar-forming population itself than systemic TGF-β inhibition, but neither treatment is completely restricted to the fibrotic response.

**Figure 6:**
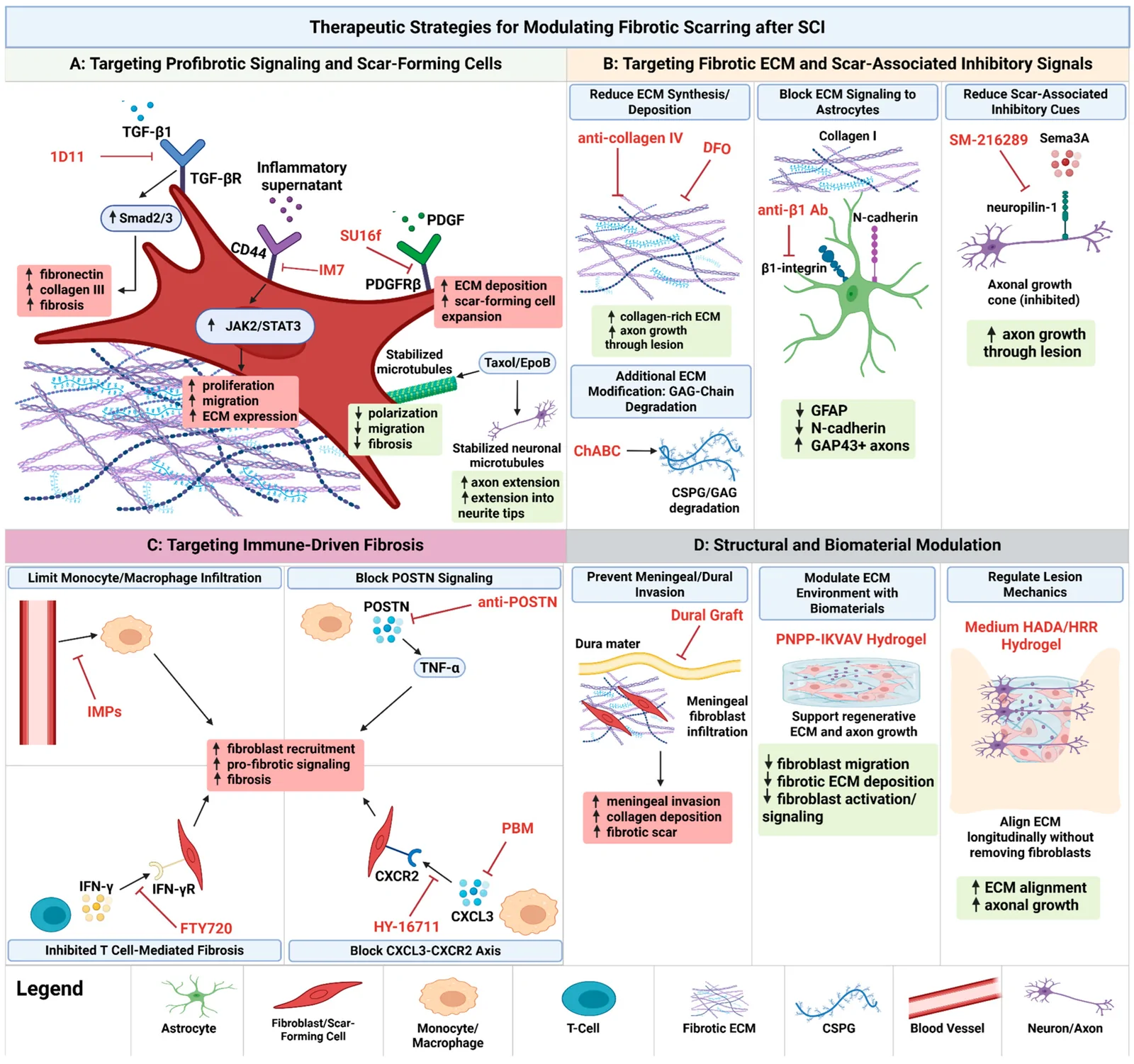
Therapeutic strategies for modulating fibrotic scarring after SCI. Many different therapeutic approaches exist for modulating scar formation, targeting different levels of signaling and repair. Some approaches shown were investigated in non-SCI CNS injury models and are included as mechanistically relevant strategies rather than SCI-validated interventions. (**A**) TGF-β1 acts through the TGF-β receptor (TGF-βR) and downstream Smad2/3 signaling, increasing fibronectin, collagen III, and fibrosis. Treatment with 1D11 reduces this response, leading to decreased Smad2/3 signaling and a resulting decrease in fibronectin, collagen III, and fibrosis. Another receptor target is CD44, which responds to inflammatory conditioned medium through upregulation of JAK2/STAT3 signaling and ultimately increases a fibroblast’s ability to proliferate, migrate, and express/deposit fibrotic ECM components. Inhibition of the CD44 receptor with IM7 reverses this cascade, reducing overall fibrosis and fibroblast scar-forming activity. Additionally, PDGFRβ binds platelet-derived growth factor (PDGF), promoting ECM deposition and scar-forming-cell expansion, the effects of which are reduced through PDGFRβ inhibition by SU16f. Additional treatments like Taxol and epothilone B (EpoB) work differently, stabilizing microtubules in both scar-forming cells and neurons to decrease fibrosis and increase axonal extension. (**B**) Another therapeutic approach focuses on the fibrotic ECM itself and the signals produced by this environment. Treatment with anti-collagen IV or deferoxamine (DFO) reduces collagen-rich ECM deposition, with increased axonal growth through the treated lesion. Type I collagen also affects neighboring astrocytes through β1-integrin signaling, which increases GFAP and N-cadherin expression and promotes a more scar-forming astrocytic phenotype. Blocking β1-integrin reduces these changes while increasing growth-associated protein 43-positive (GAP43^+^) axons. Sema3A represents another inhibitory factor in the scar, binding neuropilin-1 on injured axons and restricting their extension. This interaction can be blocked with SM-216289, leading to increased axonal growth through the lesion. Additionally, chondroitinase ABC (ChABC) modifies the ECM by degrading GAG chains associated with CSPGs, reducing another source of inhibition within the scar environment. (**C**) Immune cells provide another target for reducing fibrosis, either by limiting how many enter the lesion or by interrupting the signals they send to scar-forming cells. Immune-modifying nanoparticles (IMPs) reduce trafficking of circulating inflammatory monocytes into the injury site, decreasing their later contribution to fibroblast recruitment and fibrosis. Anti-POSTN treatment works through a different immune interaction, blocking POSTN signaling that normally promotes monocyte/macrophage recruitment and TNF-α production, followed by increased proliferation of scar-forming cells. T cells can also promote fibrosis through interferon gamma (IFN-γ) signaling to the IFN-γ receptor (IFN-γR) on scar-forming cells. Fingolimod (FTY720) reduces T-cell trafficking into the spinal cord and therefore decreases this IFN-γ-associated response. A final immune-targeted approach comes from macrophage-derived C-X-C motif chemokine ligand 3 (CXCL3), which acts through C-X-C motif chemokine receptor 2 (CXCR2) on fibroblasts to increase migration. Photobiomodulation (PBM) reduces CXCL3 expression in macrophages, while HY-16711 directly inhibits CXCR2, with both reducing the resulting fibroblast migratory response. (**D**) Structural and biomaterial-based treatments can also change fibrosis by altering the environment where the scar develops instead of directly blocking one signaling pathway. Following disruption of the dura, connective tissue can continue entering the injury site and contributing to collagen deposition, so placement of a dural graft helps restore this physical barrier and limits continued invasion into the lesion. Taking a different approach, the thermoresponsive poly(N-isopropylacrylamide) (PNIPAM)-based hydrogel containing the laminin-derived Ile-Lys-Val-Ala-Val (IKVAV) peptide, referred to as PNPP-IKVAV, is placed directly into the injury site, where reductions in fibroblast migration, fibrotic ECM deposition, and fibroblast activation are seen. The mechanical properties of the implanted material can matter as well. HADA/HRR hydrogels were tested at different mechanical strengths, with the Medium condition most closely matching the native spinal cord. Rather than simply reducing the fibroblast population, fibroblasts remained within this condition but became more longitudinally organized along with the surrounding ECM, which was accompanied by greater axonal growth through the aligned matrix. Therefore, fibrosis can be therapeutically changed not only by reducing the amount of scar that forms, but also by changing the cells and signals that drive it and how the remaining scar is organized.

Another approach shifts from blocking receptors to altering the cytoskeletal machinery fibroblasts use to migrate and build the scar, directly affecting fibrosis without directly knocking out a fibroblast receptor. Hellal et al. delivered Taxol to T8 rat dorsal hemisection SCI models through an intrathecal catheter, with low doses reducing fibronectin, laminin, and collagen IV [64]. No changes were observed in lesion size or astrocytic compaction around the injury, suggesting it as a treatment to reduce the fibrotic component at the injury site while allowing wound containment. Mechanistically, Taxol interfered with TGF-β/Smad2/3 signaling and reduced TGF-β1-induced meningeal-cell migration, fibronectin production, and GAG/CSPG accumulation, indicating an overall change in fibroblast migration and ECM production rather than a change in one extracellular ligand. In terms of axonal growth, Hellal et al. found that more conditioned sensory axons extended through the injury site following Taxol treatment, providing evidence that reducing scarring alongside changes in neuronal microtubules could support growth of injured axons [64]. Greater numbers of 5-HT^+^ fibers were also observed caudal to the lesion, although this increase did not independently distinguish regeneration from sprouting of serotonergic fibers that may have been spared during injury. The treated animals also demonstrated improved gridwalk performance at later time points, but the behavioral findings could not establish how much of this recovery resulted from the observed axonal growth. The treatment, however, also acts directly on the neurons by stabilizing neuronal microtubules and promoting intrinsic growth, supporting this functional recovery as a combination of neuronal growth activation and fibrotic reduction with neither being the sole experimentally derived cause. The delivery of this drug remained an issue as well, since Taxol has limited CNS and blood–brain barrier (BBB) penetration. This established a stronger follow-up in the form of epothilone B (EpoB), a treatment Ruschel et al. tested due to it being a BBB-permeable microtubule-stabilizing drug [65]. They found that systemic intraperitoneal (IP) injections at 0.75 mg/kg on days 1 and 15 after SCI reduced fibronectin and laminin^+^ scar tissue as well as CSPG deposition, while the lesion area and astrogliosis remained similar to the control models. Mechanistically, EpoB disrupted the polarization and directed migration of meningeal fibroblasts, showing that its mechanism of inhibition resulted from changes in microtubule organization rather than reduced proliferation. EpoB had an opposing effect in neurons, as it promoted coordinated microtubule polymerization into neurite tips, supporting growth of neurons through inhibitory substrates including CSPGs, Nogo-A, and Sema3A, providing an example of a drug acting differently on fibroblasts and neurons. Beyond the effects on fibroblast migration, Ruschel et al. also examined the response of injured axons following EpoB treatment [65]. Through in vivo imaging, they found fewer dystrophic retraction bulbs and reduced axonal dieback, while dorsal-column tracing following transection provided additional evidence of regeneration rather than simply preservation of injured fibers. The authors also observed increased serotonergic 5-HT^+^ fibers caudal to the lesion, accompanied by improvements in walking, including stride length, gait regularity, and hindpaw positioning. However, the increased serotonergic innervation alone could not determine whether these fibers had regenerated or sprouted from spared populations. To further support these findings, Ruschel et al. chemically ablated serotonergic innervation, which abolished the functional improvements, supporting a role for restored serotonergic circuitry in recovery after SCI [65]. Therefore, microtubule stabilization provides a way to reduce fibrotic scar formation by altering fibroblast migration while also promoting neuronal growth, with this same dual effect making it difficult to determine how much of the observed functional recovery comes specifically from reduced fibrosis rather than the direct effects on regenerating axons (Figure 6A).

### 5.2. Reducing Fibrotic ECM Deposition and Scar-Associated Inhibitory Signaling

Rather than modulating the signaling pathways driving fibroblast behavior, another approach to attenuating the scar sought to reduce the production of fibrotic ECM products without eliminating the fibroblast population. Stichel et al. provided an early CNS-based study using mechanical transection of the postcommissural fornix in adult rats, utilizing anti-collagen IV antibodies and α,α′-dipyridyl (a collagen biosynthesis inhibitor), where they found a subsequent reduction in dense collagen IV-containing fibrotic scar tissue and axotomized fibers extending beyond the injury site [4]. Some regenerating fibers were able to reach and reinnervate the mammillary body, providing a non-SCI proof-of-concept for this type of therapeutic approach. More recently, Vogelaar et al. enhanced this same general idea with deferoxamine mesylate (DFO), an iron chelator that targets collagen synthesis at the iron-requiring prolyl-4-hydroxylase step [66]. In vitro, they found that DFO reduced scar-like cluster number/area, collagen IV, and other scar-associated ECM components. Furthermore, DFO did not significantly reduce fibroblast proliferation, supporting its effect being attributed to matrix production targeting rather than depletion of fibroblasts. DFO also increased neurite growth on the observed scar-like clusters but did not significantly increase neurite growth on the cluster-free cell layers, supporting an effect on the inhibitory scar environment rather than a direct neurite-facilitative growth effect. They further tested DFO in vivo in rat SCI, where local infusion was associated with reductions in collagen IV deposition and lesion size as well as increases in spared tissue, CST and calcitonin gene-related peptide (CGRP)^+^ axons within the lesion, and improved Gridwalk performance for the right hindlimb at 2, 6, 8, and 10 weeks, with the difference no longer significant at later time points. In terms of limitations, DFO is not specific to collagen synthesis, since iron chelators may also have antioxidant and neuroprotective effects in off-target systems. Additionally, since DFO-treated animals also showed greater tissue sparing, the increased presence of CST- and CGRP-positive axons within the lesion could not be attributed exclusively to regeneration of previously injured fibers. The improvements in gridwalk performance may therefore reflect a combination of changes in axonal growth, tissue preservation, and the surrounding injury environment rather than reduced collagen deposition alone. Therefore, targeting collagen synthesis can reduce the physical component of the scar without directly eliminating fibroblasts, but the matrix reduction alone does not fully restore function.

Targeting interactions between the deposited ECM and surrounding cells can also provide another therapeutic strategy. Hara et al. examined this principle in mouse contusion SCI, where type I collagen accumulated during the scar-forming phase and interacted with reactive astrocytes at the lesion boundary [30]. As previously described, collagen I promoted the transition of these astrocytes toward a more scar-forming phenotype through β1-integrin and N-cadherin-associated signaling. Hara et al. disrupted this interaction using an anti-β1-integrin antibody during days 9-13 post-SCI, finding reduced N-cadherin and GFAP expression and increased growth-associated protein 43-positive (GAP43+) axons at and past the injury site alongside improved locomotor recovery [30]. However, the treatment cannot be attributed specifically to blocking collagen I signaling because β1-containing integrins can interact with multiple extracellular ligands, meaning the results still support the broader idea that the effects of the matrix can be modified by preventing it from driving restrictive changes in neighboring astrocytes.

Another source of signaling inhibition arises from the previously mentioned Sema3A mechanism hindering axonal regrowth. Kaneko et al. treated adult rats with complete SCI using the Sema3A inhibitor SM-216289 directly at the injury site for 4 weeks through an osmotic minipump, which temporally matched the period when Sema3A expression remained elevated after injury [67]. By directly acting on Sema3A and interfering with its binding to neuropilin-1, SM-216289 increased neurofilament-positive axons, GAP43^+^ growth-associated axons, and CGRP^+^ axons within the injury site, with more 5-HT^+^ fibers also extending across the lesion. This connects to the previous finding that increasing intrinsic neuronal growth signaling alone was not enough to overcome Sema3A-rich fibrotic scar regions, whereas inhibiting Sema3A itself changed the inhibitory environment. Mechanistically, SM-216289 acted relatively selectively against Sema3A rather than broadly blocking other inhibitory signals such as Nogo-A, myelin-associated glycoprotein, oligodendrocyte myelin glycoprotein, Sema3B, or Sema3F. However, Sema3A inhibition also produced several additional effects, including altered Schwann-cell migration, increased Schwann-cell-mediated myelination, reduced apoptosis, and increased angiogenesis, broader effects that indicate the benefits likely reflect more than the reduction in Sema3A-mediated axonal inhibition alone. These changes were accompanied by functional improvements, with hindlimb Basso–Beattie–Bresnahan locomotor scores beginning to improve around 5 weeks and remaining higher at later observations. Retransection of the lesion reversed the recovered function, supporting that the improvement depended on axons crossing the lesion rather than only changes in circuitry below the injury. Furthermore, disrupting 5-HT fibers also substantially reduced recovery, emphasizing the importance of restored serotonergic signaling in functional recovery. Based on these findings, the fibrotic scar does not necessarily have to be removed to reduce its regenerative restrictions, as changing a single inhibitory signal within that environment was enough to support greater axonal growth across the lesion and improve functional recovery.

Besides the more classical fibrotic scar ECM components that have been therapeutically modulated, some targets exist beyond solely the fibrotic response. Specifically, CSPGs strongly contribute to the inhibitory environment in both fibrotic and glial scars, with many attributable studies exclusively focusing on the glial scar component. Bradbury et al. used Chondroitinase ABC (ChABC), a bacterial enzyme that degrades GAG chains in CSPGs, to inhibit CSPG hindrance within the glial scar [68]. Following treatment, improved axonal regeneration, plasticity, and functional recovery were observed. However, the connection to fibrotic scarring comes from a brain injury experiment performed by Li et al., where different enzymes were compared with different GAG specificities: ChABC degrades both chondroitin sulfate (CS) and dermatan sulfate (DS), Chondroitinase B (ChB) preferentially degrades DS, and Chondroitinase AC (ChAC) targets CS [69]. Following injury, ChABC and ChB were found to suppress fibrotic scar formation, while ChAC did not substantially reduce scar formation even though it promoted axonal regeneration. These findings suggested DS was more closely associated with fibrotic scar formation, whereas CS contributed more strongly to axonal inhibition. Overall, GAG-chain-targeted enzymatic activity shows another way the ECM can be modified, but its direct role in SCI fibrotic scar modulation specifically rather than glial scar activity remains less established (Figure 6B).

### 5.3. Modulating Immune-Driven Fibroblast Recruitment and Profibrotic Signaling

Based on the mechanisms described previously, the recruitment of fibroblasts is highly dependent on immune-cell input. To characterize the role of macrophages in immune-fibrotic signaling, Zhu et al. used clodronate liposomes to deplete hematogenous macrophages after SCI, leading to reductions in *Col1a1^+^* fibroblast density, basal-lamina organization around the fibrotic scar, and chronic fibrotic scar size, alongside increases in neurofilament-positive axonal growth within the fibrotic region [25]. Despite the increased axonal growth, the treated animals did not show improved open-field locomotor recovery, which emphasizes that a clear distinction existed between neuroprotection, axonal sprouting, and true axonal regeneration. In other words, we should be aware that increased expression of axonal markers does not necessarily demonstrate genuine axonal regeneration; furthermore, enhanced axonal growth does not necessarily indicate the establishment of functional neuronal connections. Analysis of the specific cytokines showed decreased *Tnfsf8*/*Tnfsf13* and increased bone morphogenetic proteins, suggesting the immune depletion changed the broader profibrotic signaling environment. Transitioning from this broad-range macrophage depletion, Jeong et al. treated SCI mice with intravenous 500 nm poly(lactide-co-glycolide) immune-modifying nanoparticles, which were taken up by circulating inflammatory monocytes through a macrophage scavenger receptor, limiting their migration to inflammatory sites [39]. Importantly, these nanoparticles did not bind resident microglia or reduce their numbers. In terms of fibrosis, fewer hematogenous monocytes entering the cord were associated with reduced fibrotic scar formation, M1-type macrophage polarization, and inflammatory cytokines while glial scarring remained similar. Additionally, CSPG accumulation was reduced, allowing increased axon density within and past the injury site and improved functional recovery after both moderate and severe SCI. These approaches show an upstream approach to reducing fibrosis by limiting the immune cells that help drive fibroblast recruitment and activation, constituting an alternative pathway that can be potentially targeted to reduce scarring.

Rather than reducing entire immune-cell populations, other approaches have targeted individual signaling pathways connecting inflammatory cells to fibrotic scar formation. Yokota et al. targeted POSTN, a mechanism previously described [40]. They observed an increase in POSTN after injury that peaked around 7 days during active scar formation in mouse T9 contusion SCI, which was predominantly associated with PDGFRβ^+^ cells that Yokota et al. called scar-forming pericytes [40]. Subsequent genetic deletion of *Postn* reduced proliferation of these cells and reduced scar formation. Mechanistically, recombinant POSTN increased monocyte and macrophage migration and increased macrophage TNF-α, the latter of which promoted proliferation of the PDGFRβ^+^ cells. Most importantly, pharmacological blocking of POSTN reduced scar formation and improved long-term functional outcomes. Further studies by Li et al. shifted the focus from macrophage signaling to a separate immune-fibroblast pathway involving T-cell-derived IFN-γ [70]. They found IFN-γ closely associated with PDGFRβ^+^ fibroblasts after mouse T10 crush SCI and identified T cells as a major source. Furthermore, the signaling significance of IFN-γ was supported by its direct injection into the uninjured spinal cord being sufficient to induce fibroblast accumulation, fibrotic scarring, and inflammation. Next, they used fingolimod (FTY720), a sphingosine-1-phosphate receptor 1 (S1PR1) modulator that reduces T-cell egress from lymph nodes and subsequent infiltration into the CNS. This involves the same broader S1P receptor family described previously, although here the relevant signaling is through S1PR1 on T cells rather than S1P3 on scar-forming cells. Daily 2 mg/kg IP FTY720 reduced T-cell infiltration and inhibited signaling between IFN-γ and the interferon gamma receptor (IFN-γR), markedly decreasing fibrotic scarring, inflammation, and lesion size while improving neuroprotection and locomotor recovery. To test whether IFN-γ suppression contributed to the anti-fibrotic effect, Li et al. added IFN-γ back into the lesion, which partially reversed the reduction in fibrotic scarring produced by FTY720 [70]. As with other non-specific treatments, the broad immunomodulatory effects of FTY720 on processes such as lymphocyte trafficking, inflammation, and lesion size provide additional reasons for recovery beyond direct inhibition of scar formation, while the overall findings demonstrate that immune cells can be targeted at specific signaling interfaces rather than by depleting the entire immune-cell population.

Whereas previous treatments target the signaling and proliferation of immune cells, other treatments can directly alter macrophage behavior. Zhang et al. treated a mouse T10 compression SCI model with 808 nm photobiomodulation (PBM), which uses low-energy laser irradiation to alter cellular activity daily for a total of 28 days [71]. This led to reduced fibrotic scar area, fibronectin, and collagen I, as well as increased βIII-tubulin^+^ area within the lesion, BMS, and Louisville Swimming Scale (LSS) scores. To determine how PBM was producing these changes in fibrosis, they next examined when macrophages and fibroblasts appeared following injury, finding that macrophages accumulated earlier while fibroblast and ECM accumulation followed, suggesting that macrophages could be affecting the later fibrotic response. Next, in vitro experimentation found that inflammatory macrophage-conditioned medium increased meningeal fibroblast migration, whereas conditioned medium from PBM-treated macrophages caused less migration, with neither condition significantly affecting fibroblast proliferation itself. By conducting RNA sequencing, inflammatory macrophages with and without PBM were compared, with C-X-C motif chemokine ligand 3 (CXCL3) emerging as one of the strongly altered cytokines between conditions. Namely, PBM reduced CXCL3 RNA, protein, and CXCL3^+^ macrophages in vivo. To test whether CXCL3 had a real causal effect on fibroblast migration, they targeted C-X-C motif chemokine receptor 2 (CXCR2), the receptor through which CXCL3 signals. By adding recombinant CXCL3 and blocking CXCR2 with HY-16711 in separate experiments, they found that fibroblast migration increased and decreased, respectively. This signaling intermediate was tested in vivo as well, where HY-16711 was given via IP injection after SCI and led to reductions in fibronectin, collagen I, and fibrotic scar area and increases in βIII-tubulin^+^ area and BMS/LSS recovery. While PBM remains an important modulator of macrophage-fibroblast signaling through the CXCL3/CXCR2 pathway, the exact upstream mechanism behind how PBM causes macrophages to reduce CXCL3 remains unresolved (Figure 6C).

### 5.4. Restoring Structural Barriers and Engineering the Lesion Environment to Modulate Fibrosis

Structural interventions can play an important role in changing how fibrotic tissue enters the injury site and organizes the ECM. Iannotti et al. directly studied repair following rat cervical spinal cord laceration through the implantation of a dural allograft along the damaged dura [72]. This graft led to reductions in connective tissue scar invasion, cystic cavity formation, post-SCI inflammation, and type IV collagen deposition in the scar with a notable improvement in cerebrospinal fluid circulation. These findings show that fibrosis can be reduced by simply restoring the physical meningeal barrier that was broken, limiting continued connective tissue invasion into the spinal cord microenvironment. Taking a different approach than physically preventing connective-tissue invasion, Jiang et al. implanted a thermoresponsive poly(N-isopropylacrylamide) (PNIPAM)-based hydrogel containing the laminin-derived Ile-Lys-Val-Ala-Val (IKVAV) peptide, referred to as PNPP-IKVAV, following rat spinal cord transection [73]. IKVAV has previously been used to promote neuronal differentiation and axonal growth. Fourteen days post-injury, prominent reductions in fibronectin and collagens I and IV were observed, showing that the hydrogel reduced the deposition of several major fibrotic ECM components rather than targeting only one matrix protein. Adjacent in vitro studies showed that IKVAV markedly reduced fibroblast migration, while separate experiments found reductions in TGF-β1-associated signaling and α-SMA, along with reductions in endoplasmic reticulum stress markers including glucose-regulated protein 78, inositol-requiring enzyme 1, X-box binding protein 1, and CCAAT/enhancer-binding protein (C/EBP) homologous protein, showing several changes in fibroblast activation in addition to migration itself. Rather than changing the biochemical components of the hydrogel, Xiao et al. tested whether the mechanical strength of the implanted material itself could change how fibroblasts organize the developing scar [50]. Following rat T10 complete transection with a 2 mm spinal cord segment removed, they implanted HADA/HRR hydrogels with Low, Medium, or High mechanical strength, with the Medium condition most closely matching the mechanical properties of the native spinal cord. The Low and High conditions developed dense and disorganized ECM, while the Medium hydrogel caused laminin, collagens I and IV, and fibronectin to organize into a more parallel arrangement running longitudinally with the spinal cord. PDGFRβ^+^/*Col1a1^+^* fibroblasts were still present within the matrix after treatment, so the effect of the mechanically matched hydrogel appears to come from changing how these cells migrated and arranged the ECM rather than removing the scar-forming population itself. Xiao et al. further identified an *Il11ra1^+^*/*Itga11^+^* fibroblast subset that was enriched in the Medium condition and associated with this aligned migration and ECM organization, with the LRP6/β-catenin/MMP7 mechanism underlying this response described previously [50]. This restructuring of the fibrotic matrix was accompanied by greater axonal growth, with Xiao et al. using retrograde tracing to examine axonal projections associated with the injured region [50]. The researchers also examined synaptic structures using electron microscopy, identifying both excitatory and inhibitory synapse-like structures within the treated tissue. These findings provided anatomical support for the reestablishment of neural connections following hydrogel treatment, although the presence of these structures did not independently determine how much of the observed functional recovery was attributable to the newly formed connections. Overall, the results suggest that biomaterial approaches do not necessarily have to remove fibrotic ECM to make the injury site more permissive, as changing the organization of the remaining matrix can also support axonal growth. Together, these studies demonstrate that structural treatments can reduce fibrosis by either limiting how connective tissue enters the lesion or changing how fibroblasts organize the matrix once they are already present (Figure 6D).

### 5.5. Translational Relevance of Preclinical Strategies for Chronic SCI

Currently available antifibrotic medications (such as nintedanib and pirfenidone) are orally administered systemic medications that can cause a multitude of side effects, depending on the drug and individual. These include gastrointestinal, dermatological, metabolic, hepatic, and other systemic adverse effects, with specific complications including diarrhea, nausea, vomiting, abdominal pain, photosensitivity, skin rashes, and elevated liver enzymes [61,74,75]. Thus, there remains an urgent need to develop more effective antifibrotic therapies with minimal or no adverse effects.

Following SCI, fibrotic scars composed of fibroblasts, pericytes, and inhibitory ECM components form at the lesion site, creating physical and biochemical barriers that restrict axonal regeneration and reconnection [35,44,76]. Commercially available antifibrotics function primarily by slowing fibroblast proliferation and the active deposition of new scar tissue. In chronic SCI, however, the fibrotic scar is already consolidated and mature. Standard antifibrotics have not been demonstrated to dissolve or reverse established, mature CNS scars [76,77]. As a result, currently available oral antifibrotics may not adequately address the mature scar present in chronic SCI. Therefore, to effectively manage chronic spinal cord lesions, future therapeutic agents must possess the capacity to (1) efficiently cross the blood–spinal cord barrier and penetrate the dense, poorly vascularized fibrotic matrix, which may present a major limitation for systemically administered antifibrotics; and (2) directly reduce or remove established fibrotic scars in chronic SCI.

## 6. Conclusions

Overall, fibrotic scarring after SCI acts as a double-edged sword, demonstrating beneficial and detrimental effects based on the timing, extent, composition, and organization of the resulting response. Early on, fibrotic scarring helps close the injury site, preventing tissue defects and helping contain inflammatory cells within the lesion, but sustained proliferative scar signaling eventually leads to an environment that is increasingly restrictive to axonal growth and neural repair. This makes fibrotic scarring especially important to study in SCI, as long-term deficits remain a major clinical problem and successful neural repair requires a sufficiently permissive environment. However, current therapeutic approaches such as antifibrotic medications have only moderate effects in slowing the progression of fibrosis and exhibit several key limitations, including an inability to reverse existing scars and adverse effects that may require temporary treatment interruptions or discontinuation. These limitations raise major unresolved questions regarding when fibrosis becomes permanent, what molecular signals turn wound healing into chronic scarring, and how to selectively target dysfunctional cells and/or fibrotic tissues. Therefore, the promising directions for future research and therapeutic development to treat fibrosis and fibrotic scars should focus not only on slowing new fibrotic deposition to delay disease progression, but also on developing targeted scar-modifying therapies with minimal off-target toxicity that can reverse established fibrotic scarring and promote tissue regeneration.

## Figures and Tables

**Table 1:** Comparison of experimental approaches used to characterize the cellular origins of fibrotic scar-forming cell populations after spinal cord injury (SCI).

Study & SCI Model	Genetic Labelling Strategy	Cellular Markers and Population	Major Findings and Limitations
Dorsal funiculus incision and dorsal hemisection [7]	Preinjury lineage tracing using glutamate-aspartate transporter (GLAST)-CreER, a tamoxifen-inducible recombination system, combined with electron microscopy	GLAST-lineage type A pericytes expressing platelet-derived growth factor receptors alpha and beta (PDGFRα/β) and cluster of differentiation 13 (CD13); distinguished from type B pericytes through differences in desmin and alpha-smooth muscle actin (α-SMA) expression	GLAST-lineage perivascular cells expanded after SCI and generated fibrotic stromal cells. Ultrastructural evidence supported their classification as a pericyte subtype, although their relationship to fibroblast populations identified in subsequent studies was not directly established.
Contusion and dorsal hemisection [3]	Col1a1-green fluorescent protein (GFP) reporter combined with neuron-glial antigen 2 (NG2)-CreER/Rosa26-tdTomato lineage tracing	*Col1a1*-expressing perivascular fibroblasts frequently expressed PDGFRβ and CD13, while the examined uninjured population lacked NG2, nestin, and α-SMA	*Col1a1*-expressing fibroblasts populated the fibrotic lesion and were distinct from traced NG2-lineage cells. Fibroblast distributions differed between contusive and penetrating injuries. The *Col1a1*-GFP reporter identified promoter activity rather than an established developmental lineage, while incomplete NG2-CreER labeling limited exclusion of other pericyte populations.
Dorsal hemisection [8]	GLAST-CreERT2-mediated lineage tracing, using a second-generation tamoxifen-inducible recombination system, and conditional manipulation of Ras signaling	GLAST-lineage type A pericytes and associated PDGFRβ^+^ scar-forming cells	Moderate attenuation of GLAST-lineage proliferation reduced fibrosis, whereas near-complete suppression impaired lesion closure. These findings established the functional importance of the targeted population but did not determine its relationship to *Col1a1/Col1a2*-expressing fibroblasts.
Transection and crush SCI [5]	PDGFRβ-CreER, *Col1a2*-CreER, *Myh11*-CreER, NG2-CreER, and *Crabp2*-CreER reporter systems combined with single-cell RNA sequencing (scRNA-seq)	*Col1a2*-expressing fibroblasts; *Crabp2^+^*/*Emb^+^* central scar fibroblasts (CE-F); *Lama1^+^*/*Lama2^+^* peripheral fibroblasts (LA-F); *Myh11*/NG2-labeled pericyte and vascular smooth muscle cell (vSMC) populations	Perivascular and meningeal fibroblasts predominated in fibrotic scar formation, whereas traced pericyte/vSMC populations contributed comparatively little in the examined models. CE-F and LA-F populations exhibited distinct spatial distributions and extracellular matrix (ECM) profiles. Their relationship to previously described GLAST-lineage type A pericytes remained unresolved.

**Table 2:** Distinct contributions of the fibrotic scar, astrocytic scar, and glial-fibrotic interface to inflammatory containment, tissue stabilization, axonal regeneration, and chronic pathological changes.

Feature/Compartment	Fibrotic Scar (Lesion Core)	Astrocytic Scar (Lesion Perimeter/Penumbra)	Glial-Fibrotic Interface (A Boundary Zone Separating the Lesion Core and Perimeter)
Primary Cellular Drivers	Perivascular fibroblasts/pericytes and infiltrating macrophages.	Reactive astrocytes and associated oligodendrocyte progenitor cells (NG2-positive).	Interdigitating astrocytes that interact directly with stromal fibroblasts.
Inflammatory Containment [33,34]	Traps and contains peripheral immune cells, including potentially destructive macrophages and neutrophils.	Forms a physical and chemical barrier that prevents the spread of damaging inflammation into healthy tissue.	Serves as a molecular checkpoint that mediates CNS-periphery immune crosstalk.
Tissue Stabilization [33,35,36]	Deposits dense collagen- and fibronectin-rich ECM to stabilize damaged tissue and vasculature.	Re-establishes a homeostatic boundary, limiting secondary tissue damage.	Acts as a mechanical transition tether that anchors the vascularized lesion core to the undamaged surrounding parenchyma.
Axonal Regeneration [36,37]	Creates strong physical and chemical barriers to axonal regeneration.	Chronically restricts axonal growth through high levels of CSPGs.	Forms a mechanical and biochemical barrier where dystrophic axon tips can become entrapped and arrested.
Chronic pathological contribution [33,36,38]	Creates dense barriers restricting the formation of anatomical bridges.	Forms a potent chemical barrier to repel and inhibit axonal growth	Maintains compartmental segregation that blocks cell migration.

**Table 3:** A temporal and spatial framework integrating reparative and pathological fibrosis based on cellular composition, ECM organization, mechanical properties, or molecular signaling profiles.

Feature	Reparative Fibrosis	Pathological Fibrosis
Primary Purpose	Restores tissue integrity following acute injury.	Driven by chronic, unremitting inflammation or injury.
Temporal Dynamic [53]	Self-limiting and acute (days to weeks).	Progressive, chronic and persistent (months to years).
Spatial Organization [53]	Highly localized and organized to the site of injury to coordinate wound-healing (acute repair).	Diffuse, disorganized, and self-perpetuating process leading to architectural destruction (chronic remodeling).
Cellular Composition [53,54]	Controlled influx of inflammatory cells, transient activation of fibroblasts, followed by timely clearance of immune cells.	Heterogeneous accumulation of senescent cells, chronically active myofibroblasts, and sustained pro-fibrotic immune subsets.
ECM Organization [55,56]	Initial chaotic deposition of collagen III, which is systematically replaced by aligned, cross-linked collagen I, matching native tissue stress lines.	Excessive, chaotic deposition of collagen, fibronectin, and proteoglycans. High density of aberrant, randomly oriented matrix fibers.
Mechanical Properties [57,58]	Transient increases in stiffness that plateau once structural integrity is achieved, avoiding mechanical feedback loops.	A self-sustaining mechanical loop in which a hyper-stiffened matrix triggers mechanotransduction pathways that further activate fibroblasts.
Molecular Signaling [59,60,61]	Transient and controlled bursts of TGF-β, PDGF, and IL-4/IL-13 signaling that are shut off by feedback antagonists (e.g., SMAD7, decorin).	Hyperactive and continuous TGF-β/SMAD, Wnt/β-catenin, and YAP/TAZ signaling without effective inhibition

## Data Availability

No new data were created or analyzed in this study. Data sharing is not applicable to this article.

## Abbreviations

5-HT	5-hydroxytryptamine
*Acta2*	actin alpha 2, smooth muscle
α-SMA	alpha-smooth muscle actin
*Atg5*	autophagy-related 5
BBB	blood–brain barrier
BDA	Biotinylated dextran amine
BMS	Basso Mouse Scale
C/EBP	CCAAT/enhancer-binding protein
CCL2	C-C motif chemokine ligand 2
CD11b	cluster of differentiation 11b
CD13	cluster of differentiation 13
CD31	platelet endothelial cell adhesion molecule-1 (PECAM-1)
CD44	cluster of differentiation 44
CD68	cluster of differentiation 68
CE-F	*Crabp2^+^/Emb^+^* fibroblast population enriched in the central scar
CGRP	calcitonin gene-related peptide
ChABC	chondroitinase ABC
ChAC	chondroitinase AC
ChB	chondroitinase B
CNS	central nervous system
*Col1a1/COL1A1*	collagen type I alpha 1 chain
*Col1a2/COL1A2*	collagen type I alpha 2 chain
*Col4a1*	collagen type IV alpha 1 chain
*Crabp2*	cellular retinoic acid-binding protein 2
CreER	tamoxifen-inducible Cre recombinase-estrogen receptor fusion system
CreERT2	second-generation tamoxifen-inducible Cre recombinase-estrogen receptor fusion system
CS	chondroitin sulfate
CS-GAG	chondroitin sulfate glycosaminoglycan
CSPG	chondroitin sulfate proteoglycan
CSPG4	chondroitin sulfate proteoglycan 4
CST	corticospinal tract
CXCL3	C-X-C motif chemokine ligand 3
CXCL4/*Cxcl4*	C-X-C motif chemokine ligand 4
CXCL12/*Cxcl12*	C-X-C motif chemokine ligand 12
CXCR2	C-X-C motif chemokine receptor 2
CXCR3/*Cxcr3*	C-X-C motif chemokine receptor 3
DFO	deferoxamine mesylate
DS	dermatan sulfate
EAE	experimental autoimmune encephalomyelitis
ECM	extracellular matrix
*Emb*	embigin
EndoMT	endothelial-to-mesenchymal transition
EphB2	ephrin type-B receptor 2
EpoB	epothilone B
*Fn1*	fibronectin 1
FnEDA	fibronectin containing alternatively spliced Extra Domain A
FTY720	fingolimod
GAG	glycosaminoglycan
GAP43	growth-associated protein 43
GFAP	glial fibrillary acidic protein
GFP	green fluorescent protein
GLAST	glutamate-aspartate transporter
HADA	hyaluronic acid-graft-dopamine
HRR	HGF-(RADA)4-DGDRGDS designer peptide
Iba1	ionized calcium-binding adaptor molecule 1
IFN-γ	interferon gamma
IFN-γR	interferon gamma receptor
IKVAV	Ile-Lys-Val-Ala-Val
*Il11ra1*	interleukin 11 receptor subunit alpha 1
IMPs	immune-modifying nanoparticles
IP	intraperitoneal
*Itga11*	integrin subunit alpha 11
JAK2	Janus kinase 2
L1	L1 cell adhesion molecule
LA-F	*Lama1^+^/Lama2^+^* fibroblast population enriched at the lesion periphery
*Lama1*	laminin subunit alpha 1
*Lama2*	laminin subunit alpha 2
LRP6	low-density lipoprotein receptor-related protein 6
LSS	Louisville Swimming Scale
LysM	lysozyme M
MBP	myelin basic protein
miR-21-5p	microRNA-21-5p
MMP7	matrix metalloproteinase 7
MS	multiple sclerosis
*Myh11*	myosin heavy chain 11
NG2	neuron-glial antigen 2
NGF	nerve growth factor
OLIG2	oligodendrocyte transcription factor 2
OPC	oligodendrocyte progenitor cell
PBM	photobiomodulation
PDGF	platelet-derived growth factor
PDGFRβ/*Pdgfrb*	platelet-derived growth factor receptor beta
PDZ	postsynaptic density protein 95/discs large/zonula occludens-1
PGP9.5	protein gene product 9.5
PI3K/Akt	phosphoinositide 3-kinase/protein kinase B
PLGA	poly(lactide-co-glycolide)
PNIPAM	poly(N-isopropylacrylamide)
PNPP-IKVAV	PNIPAM-based hydrogel containing IKVAV
POSTN/*Postn*	periostin
pSmad2	phosphorylated Smad2
PTPσ	protein tyrosine phosphatase sigma
RNA	ribonucleic acid
Rosa26	ROSA26 reporter locus
S1P	sphingosine-1-phosphate
S1P3	sphingosine-1-phosphate receptor 3
S1PR1	sphingosine-1-phosphate receptor 1
SCI	spinal cord injury
scRNA-seq	single-cell RNA sequencing
Sema3A	semaphorin 3A
SMI-312	Antibody recognizing phosphorylated neurofilament proteins
SPHK1	sphingosine kinase 1
*Spp1*	secreted phosphoprotein 1
STAT3	signal transducer and activator of transcription 3
*Tagln*	transgelin
TAZ	transcriptional coactivator with PDZ-binding motif
TCF/LEF	T-cell factor/lymphoid enhancer factor
tdTomato	Tandem-dimer Tomato fluorescent reporter protein
TGF-β	transforming growth factor beta
TGF-β1/*Tgfb1*	transforming growth factor beta 1
TGF-βR	transforming growth factor beta receptor
TGFBR2/*Tgfbr2*	transforming growth factor beta receptor 2
Thy-1	thymocyte differentiation antigen 1
TNF-α	tumor necrosis factor alpha
*Tnfsf8*	tumor necrosis factor superfamily member 8
*Tnfsf13*	tumor necrosis factor superfamily member 13
TOPgal	TCF/LEF optimal promoter/β-galactosidase reporter
vSMCs	vascular smooth muscle cells
YAP	Yes-associated protein
